# A Computerized Analysis with Machine Learning Techniques for the Diagnosis of Parkinson’s Disease: Past Studies and Future Perspectives

**DOI:** 10.3390/diagnostics12112708

**Published:** 2022-11-05

**Authors:** Arti Rana, Ankur Dumka, Rajesh Singh, Manoj Kumar Panda, Neeraj Priyadarshi

**Affiliations:** 1 Computer Science & Engineering, Veer Madho Singh Bhandari Uttarakhand Technical University, Dehradun 248007, Uttarakhand, India; 2 Department of Computer Science and Engineering, Women Institute of Technology, Dehradun 248007, Uttarakhand, India; 3 Department of Computer Science & Engineering, Graphic Era Deemed to be University, Dehradun 248001, Uttarakhand, India; 4 Division of Research and Innovation, Uttaranchal Institute of Technology, Uttaranchal University, Dehradun 248007, Uttarakhand, India; 5 Department of Project Management, Universidad Internacional Iberoamericana, Campeche 24560, Mexico; 6 Department of Electrical Engineering, G.B. Pant Institute of Engineering and Technology, Pauri 246194, Uttarakhand, India; 7 Department of Electrical Engineering, JIS College of Engineering, Kolkata 741235, West Bengal, India

**Keywords:** Parkinson’s disease, artificial neural network, machine learning, deep learning, diagnosis, MRI

## Abstract

According to the World Health Organization (WHO), Parkinson’s disease (PD) is a neurodegenerative disease of the brain that causes motor symptoms including slower movement, rigidity, tremor, and imbalance in addition to other problems like Alzheimer’s disease (AD), psychiatric problems, insomnia, anxiety, and sensory abnormalities. Techniques including artificial intelligence (AI), machine learning (ML), and deep learning (DL) have been established for the classification of PD and normal controls (NC) with similar therapeutic appearances in order to address these problems and improve the diagnostic procedure for PD. In this article, we examine a literature survey of research articles published up to September 2022 in order to present an in-depth analysis of the use of datasets, various modalities, experimental setups, and architectures that have been applied in the diagnosis of subjective disease. This analysis includes a total of 217 research publications with a list of the various datasets, methodologies, and features. These findings suggest that ML/DL methods and novel biomarkers hold promising results for application in medical decision-making, leading to a more methodical and thorough detection of PD. Finally, we highlight the challenges and provide appropriate recommendations on selecting approaches that might be used for subgrouping and connection analysis with structural magnetic resonance imaging (sMRI), DaTSCAN, and single-photon emission computerized tomography (SPECT) data for future Parkinson’s research.

## 1. Introduction

Parkinson’s disease, commonly known as Tremor, is affected by a diminution in dopamine levels in the brain, which damages a person’s motion functions, or physical functioning. It is one of the world’s most common diseases. Intermittent neurological signs and symptoms result from these lesions, which become worse as the disease progresses [1]. Because aging causes changes in our brains, such as loss of synaptic connections and changes in neurotransmitters and neurohormones, this condition is more frequent among elders. With the passage of time, the neurons in a person’s body begin to die and become inimitable. The consequences of neurological problems and the falling dopamine levels in the patient’s body show gradually, making it difficult to detect until the patient’s condition requires medical treatment [2]. However, the symptoms and severity levels are different for individuals. Major symptoms of this disease are deficiency in speech, short-term memory loss, loss of balance, and unbalanced posture [1].

Every year, 10 million cases of this disease are registered worldwide, as per the WHO report. The chance of developing this disease rises with age; currently, there are 4% of sufferers worldwide under 50 years of age [2,3,4]. This disease is the highly widespread neurodegenerative disease in the world, after AD [3,4] impacting millions of people [5]. The therapy for this disease is still in its initial stages, and doctors can only assist patients in alleviating the symptoms of the disease [6]. However, there are no definite diagnostics for this disease, and the diagnosis is largely dependent on the medical history of the patient [1]. Invasive procedures are typically used for diagnosis and therapy, which are both expensive and demanding [7].

Traditionally, motor symptoms have been used to make the PD diagnosis. Although the cardinal signs of PD have been established in clinical assessments, the majority of the rating scales used to determine the severity of the disease have not been thoroughly examined and validated [8]. Despite the fact that non-motor symptoms, such as cognitive and behavioral abnormalities, sleep disorders, and sensory abnormalities like olfactory dysfunction, are common in many patients before the onset of PD [8,9,10,11,12,13,14], they lack specificity, are challenging to diagnose, and vary from patient to patient [15]. Therefore, non-motor symptoms cannot yet be utilized to diagnose PD on their own [16], despite the fact that some of them have been used as supportive diagnostic criteria [17].

In recent years, ML has emerged as a role model in the healthcare industry. As its name suggests, ML enables a computer program to acquire knowledge and derive valuable information from data with little to no human intervention. Numerous data modalities, such as movement data (such as handwriting [18,19]) or gait [20,21,22], neuroimaging [23,24,25], voice [26,27], cerebrospinal fluid [28,29] (CSF), cardiac scintigraphy [30], serum [31], and optical coherence tomography (OCT) [32] have been subjected to the application of ML models for the diagnosis of PD. In order to diagnose PD, ML also enables the combination of other modalities, such as MRI and SPECT data [33,34]. We can therefore utilize ML techniques to discover pertinent aspects that are not often used in the clinical diagnosis of PD and rely on these alternative metrics to diagnose PD in preclinical stages or atypical forms.

### 1.1. Artificial Intelligence and Machine Learning-Based Detection of Parkinson’s Disease

Over the past few decades, researchers have looked at a new way of detecting this disease through ML Techniques, a subset of AI. Clinical personnel might better recognize this disease patients by combining traditional diagnostic indications with ML.

As walking is the most common activity in every person’s day-to-day life, it has been linked to physical as well as neurological disorders. This disease, for example, has identifiable using gait (mobility) data. The Gait analysis approaches offer advantages such as being non-intrusive and having the future to be extensively used in residential settings [35]. Few sections of researchers have attempted to combine ML methods to make the procedure autonomous and possible to do offline [36].

Furthermore, persons with a subjective disease in its early stages might cause speech problems [37]. These include dysphonia (weak vocal fluency), echoes repetitious (a tiny assortment of audio variations), and hypophonia (vocal musculature disharmony) [7,38]. Information from human aural emissions might be detected and evaluated using a computing unit [39].

### 1.2. Research Problem and Motivation of Current Systematic Review

Presently, diagnosing PD in the early stage is quite challenging for the medical fraternity. Even if their health deteriorates, people can enhance their quality of life if they receive an early diagnosis. It’s challenging since PD symptoms coincide with those of other diseases, making it possible for PD to go unrecognized or, worse, to receive a misdiagnosis. Another issue is that, typically, the diagnosis of PD requires a number of steps, including gathering a thorough neurological history from the patient and examining their motor abilities in various environments.

The main purpose of this study is to summarize and assess the review of AI algorithms, data acquisition methods, and applications of AI in the diagnosis of subjective diseases and challenges. The majority of recent studies deal with the homo dataset (text, speech, video, or image). Many researchers applied voice data since they can only use so much data (single data type). Problems with dataset modification and multi-data handling procedures have been highlighted in the suggested study. The effectiveness of disease prediction is regulated as a result of the examination of a particular dataset. More real-time solutions are made possible by the use of ML-based techniques for multivariate data processing. Multi-variatevocal data analysis (MVDA) is driven to provide multiple dataset attribute-based PD identification utilizing ML approaches. This study examines the potential for improving multi-variate and multimodal data processing, which aids in raising the disease detection rate. The existing research simultaneously concentrated on various ML-based such as support vector machines (SVM), naïve Bayes (NB), K nearest neighbor (K-NN), and artificial neural network (ANN) evaluations of Parkinson’s data based on voice features. A larger number of patients were selected for the study of Parkinson’s data in the experimental works of current systems. The MVDA employs extensive datasets and ML approaches to improve disease identification based on these works. The incorporation of numerous patients’ multi-variate acoustic characteristics in the proposed MVDA is encouraged. The subjective disease has been diagnosed with the help of proposed ML techniques under the MVDA system.

### 1.3. Contribution

This research article covers the techniques of ML which are implemented in the auditory analysis of speech to diagnose this disease. The benefits and shortcomings of these algorithms in detecting the disease are thoroughly contrasted, and existing comparative studies’ potential drawbacks are explored. The main contribution of this paper is as follows:

- In this paper, we reviewed the significant statistics and relevant information collected from 217 articles (from various resources) published from 2015–2022 on the diagnosis and classification of PD.
- The fundamental discussion on AI and ML with their significance in the field of medical healthcare.
- In order to improve the prediction of PD, we also present recommendations for future perspectives to help researchers and scholars in recognizing various plausible paths for them to work in the future.

### 1.4. Structure of Proposed Work

**Figure 1:**
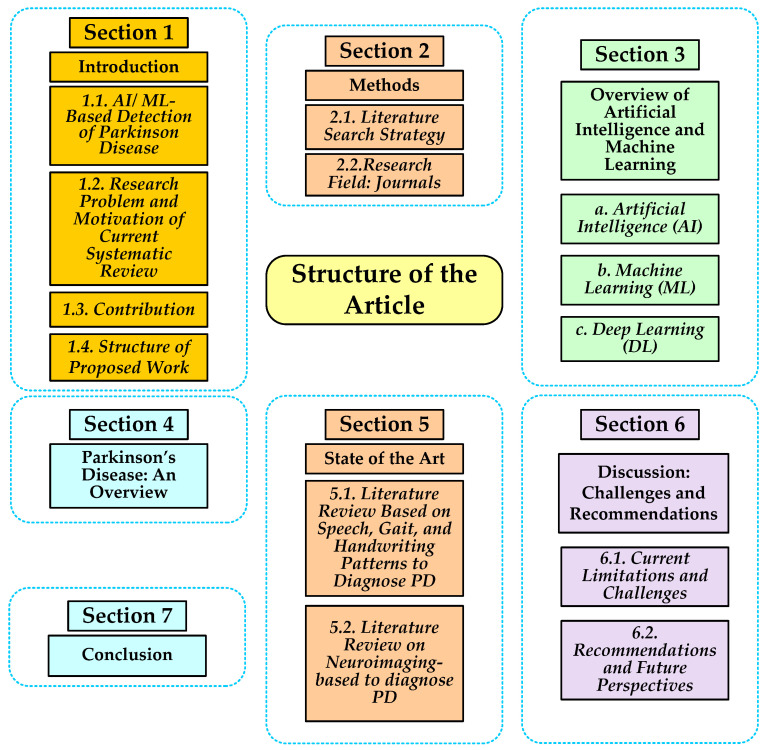
Structure of Proposed Work.

The structure of the study is as follows (Figure 1): Section 2 describes the methods for the literature search strategy. Section 3 discusses the overview of AI, ML, and DL techniques. Section 4 defines an overview of Parkinson’s disease. Section 5 illustrates the state of the art. Section 6 discusses the current limitations as challenges and future perspectives as recommendations. Finally, Section 7 defines the conclusion.

## 2. Methods

**Figure 2:**
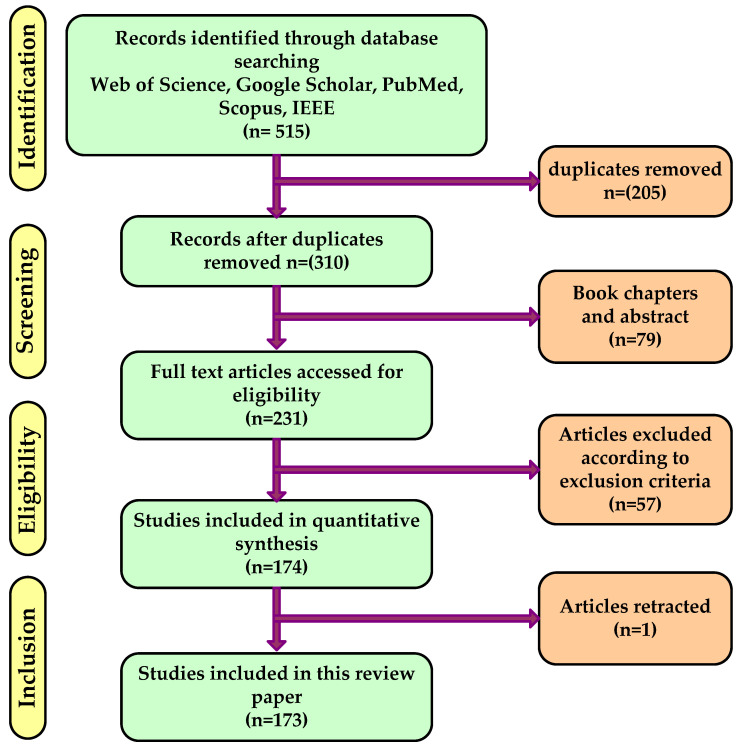
PRISMA criteria.

The present literature evaluation was carried out in a systematic manner that is generally in accordance with the most recent PRISMA criteria [40] as shown in Figure 2. By using the PRISMA technique, the authors can easily evaluate various studies as well as make decisions about the criteria for the final selection of the studies, the search strategy and data sources, and inclusion and the exclusion process.

### 2.1. Literature Search Strategy

Firstly, the most appropriate literature databases were chosen to review the research article. Web of Science, Google Scholar, PubMed, Scopus, and IEEE are five significant databases that were examined to determine and screen relevance. A total of 303 records were identified through a web of science database searching by using the keyword “Parkinson’s Disease and Artificial Intelligence” where 147 were review articles, 40 were proceeding papers, 7 were from early access, 109 were from open access, and 312 articles were found through other sources.

**Figure 3:**
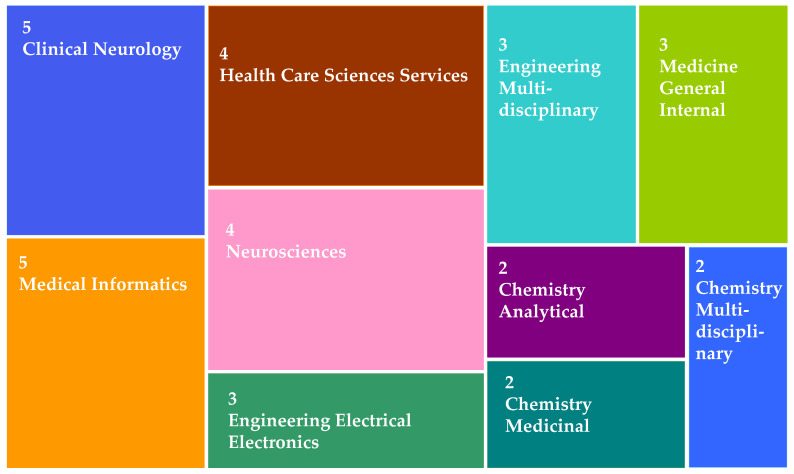
Distribution of journals from Web of Science from Core Collection.

For choosing the relevant articles, the most appropriate keywords were selected, to ensure that papers that would address the presented research topics were included. The keywords such as PD, AI, ML, and ANN are combined using the logical expression “AND”. After making multiple revisions to assure the inclusion of all the methodologies and techniques, the final technical keywords were chosen based on various approaches covered in earlier review articles. The articles considered in this study were published in journals, book chapters, abstract meetings, or conference proceedings from January 2015 to September 2022, and all the articles are written in English. After applying the logical operation in the keywords (“Parkinson’s Disease” AND (Artificial Intelligence AND Machine Learning AND Artificial Neural Network OR Deep Learning) AND (gait analysis AND voice AND rigidity AND olfactory)) the total number of 33 research papers were found from various reputed journals as shown in Figure 3. After refining the articles from the web of science core collection, the next search query was submitted to the Google scholar database by using the same pattern and search strategy from 2015 to 2022 and a total 67 results were shorted out. By using the same pattern and strategy, the queries were submitted to the rest of the databases.

### 2.2. Research Field: Journals

**Figure 4:**
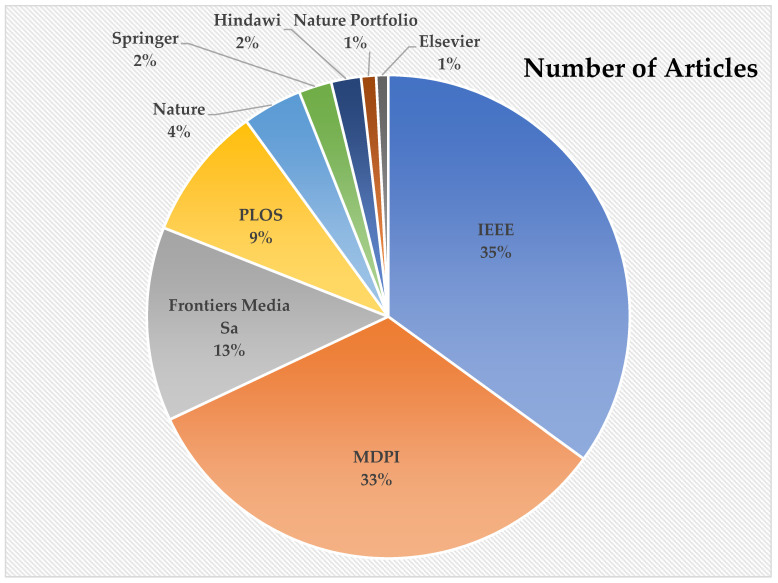
Distribution of articles based on publishers.

In this section, we organize a methodical study of the diagnosis of subjective disease using the AI/ML/DL approaches by analyzing 246 articles published during 2015–2022. The prominent journals publishing on the diagnosis of PD using different AI and ML approaches are shown in Table 1. In order to conduct this analysis, we analyzed the research papers from the MDPI (81), IEEE (86), Frontiers Media SA (31), PLOS (23), Nature (9), Springer (6), Hindawi (5), Nature Portfolio (3), and Elsevier (2), as shown in Figure 4. The research that has been included in this review shows that relevant information about the motor and non-motor symptoms of PD may be retrieved using feature selection approaches with the aid of ML algorithms, enabling clinicians to make decisions based on the given dataset. Table 2 summarizes the scope of the review article to diagnose PD (2015–2022) from the Web of Science Database.

## 3. Overview of Artificial Intelligence, Machine Learning, and Deep Learning

This section will provide a basic overview of how AI, ML, and DL work, the most popular AI, ML, and DL algorithms, and the various technologies that may be used to gather data to feed into these algorithms.

(a) Artificial Intelligence

**Figure 5:**
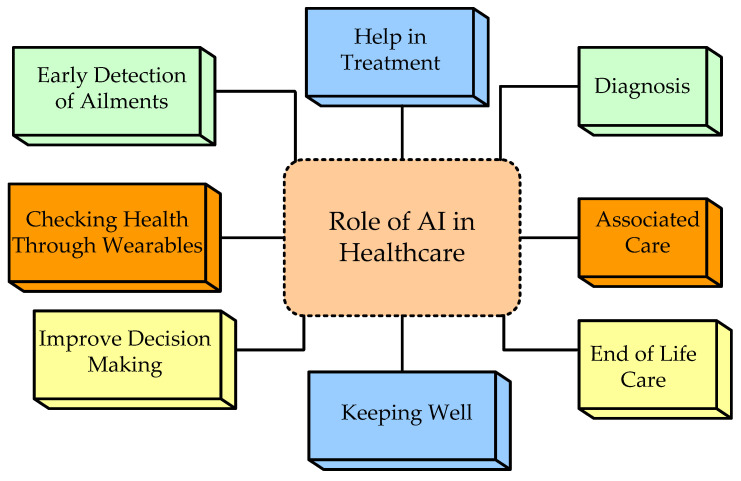
Role of artificial intelligence in healthcare.

AI is the area of computer science that aims to reproduce human intellectual abilities in machines, especially computer systems. Some particular applications of AI include expert systems, ML, speech recognition, and natural language processing (NLP) including mundane tasks, formal tasks, and expert tasks [68]. Figure 5 represents the role of AI in medical healthcare.

For the past 50 years, AI in healthcare has been primarily focused on the diagnosis and treatment of diseases. Early rule-based systems might have properly diagnosed and treated diseases, but they were not entirely adopted in clinical practice. In addition to having a less-than-perfect connection with clinical workflows and health record systems, they were not significantly more accurate at diagnosing than humans.

(b) Machine Learning

**Figure 6:**
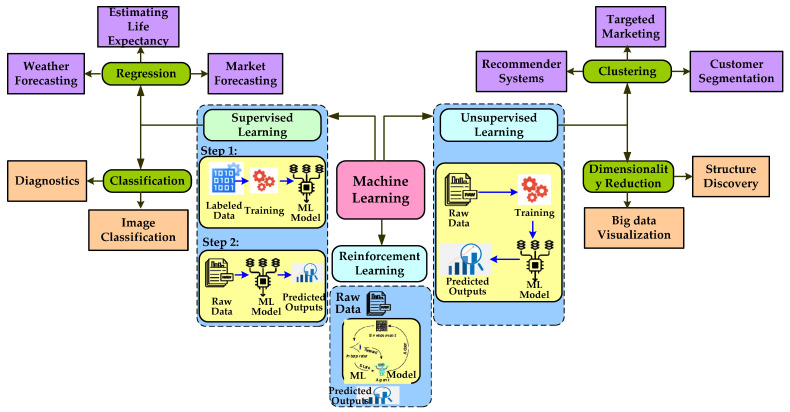
Categories of machine learning algorithm.

ML is a subset of AI in which the machine constructs a prediction model using historical data from its past experiences, predicts the outcome for new data, and becomes better at doing so. It is different from traditional programming. In traditional programming, rules are not explicitly learned from the data; rather, they are written in a computer language. Unlike traditional programming, ML creates predictive models using data that are then applied to predictions using data that have not yet been seen. Due to the intricacy of the code, it might be highly challenging to design a rule-based program for some problems. In these situations, ML can be employed if there are enough data available that is pertinent to the problem under consideration [69]. ML can be classified into supervised learning (SL), unsupervised learning (UL), and reinforcement learning (RL) as shown in Figure 6. In SL, sample-labeled data are given to the ML model as a training dataset, and on the basis of that, it predicts the outcome [70]. In UL, the ML model is trained with a collection of unlabeled, unclassified, or uncategorized data, and the algorithm is required to respond independently to that data. The main objective of UL is to reorganize the input data into new features or a collection of objects with related patterns [71]. RL is a form of ML approach where an intelligent agent (computer program) interacts with the environment and learns to function within that. By accumulating the greatest benefits over time, the goal of RL is to identify the “policy” that works best. The policy decides what should be conducted in a certain circumstance [72].

(c) Deep Learning

**Figure 7:**
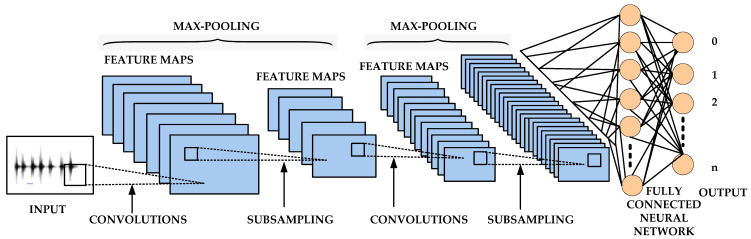
Representation of deep learning model using CNN and ANN.

Deep structured learning is a subfield of ML methods based on ANN with representation learning. DL has enormous potential in the fields of healthcare and medicine due to the sheer amount of data being produced (150 exabytes, or 10^18^ bytes, in the United States alone, expanding 48% annually) as well as the rising number of medical equipment and electronic medical record systems [73]. DL algorithms are often effective with higher dimensional data, such as audio, video, and images. DL algorithms are created dynamically to run across several layers of neural networks, which are nothing more than a collection of decision-making networks that have been pre-trained to do a certain task. Each of them is then moved on to the following layer after being run through basic layered representations. On datasets containing hundreds of features or columns, however, most ML techniques are enhanced to perform quite well. ML often fails to detect a straightforward image with dimensions of 800 × 1000 in RGB, regardless of how organized or unstructured the data set is. A standard ML system would find handling such depths to be rather impractical [74]. Recently DL techniques have been introduced for the automatic detection and categorization of PD using speech patterns and handwriting patterns recorded by a smart pen [75,76,77,78]. Examples of DL include CNN and ANN. ANNs, which are networks of computing units that mimic the functioning of biological neural networks, are frequently employed for a variety of tasks, including classification, regression, and time series analysis. They are made up of several processing layers, with the input samples being held in the first layer and the prediction being provided in the last layer. Additionally, CNN is an adaptation of ANN that uses spatial filters (convolutional layers) to extract the textures, patterns, and intrinsic properties of images [79]. By subsampling the features obtained by the convolutional layers, pooling is also used to provide reliable features as shown in Figure 7. The main advantage of the aforementioned techniques is their capacity to automatically learn EEG parameters and detect anomalies based on those features.

## 4. Parkinson’s Disease: An Overview

**Figure 8:**
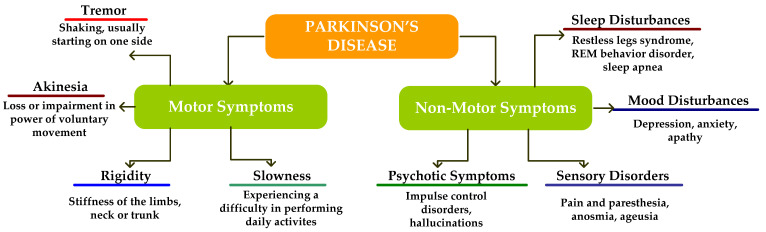
Categorization of Parkinson’s disease.

The brain’s substantianigra area experiences nerve cell declension, which causes PD. A neurotransmitter called dopamine, which was created by nerve cells is produced by this region of the brain. Dopamine’s function is to serve as a connection between the brain and the parts of the sensory organs that control and direct bodily movements [80]. When these neurons die or are harmed, there is less dopamine in the brain. This suggests that the area of the brain responsible for controlling movement is dysfunctional, which causes sluggish, undesired, and erratic motions of the physical parts [81]. Nerve cell degeneration happens gradually. PD symptoms start to manifest after around 80% of the nerve cells in the substantianigra are destroyed [82]. Currently, a combination of ecological factors and genetic abnormalities are thought to be the disease’s etiology. Although some inherited factors have been shown to increase a person’s chance of developing PD, it is unclear how these factors make some people more sensitive to the disease [83]. PD can occur in families because the dysfunctional genes are passed down from parents to children. But given the condition, this is an uncommon type of legacy. Some specialists claim that ecological factors may potentially increase a person’s chance of developing PD [84]. The AI-based algorithm may classify people as having PD or not (non-PD) based on their motor symptoms (risk factors). The training model may be developed using the dataset, which was created while evaluating the patients. Numerous PD risk factors, including both motor and non-motor variables, are formed. Although the symptomatic data cannot be statistically resolved, it is possible to improve PD detection by using an ML or DL to better grasp both data classes [85]. In-feature AI is the best choice to accurately predict PD while examining the symptomatic biology of the disease. Due to the progressive nature of PD, its severity and status have been assessed using the following subjective and varied assessment systems [86,87,88,89,90,91]—MoCA, screening questionnaire, GDS, RBD, UPDRS, STAI, PIGD score, SCOPA-AUT, and MMSE, [92]. Figure 8 depicts the category of PD.

Parkinsonian gait or festinating gait is the type of gait exhibited by patients with PD. Parkinson’s patients frequently experience feeling as though they are “locked in place” when taking a stride or turning, which raises the possibility of falling. This disorder is brought on by a dopamine shortage in the basal ganglia circuit, which results in motor impairments. Despite the fact that PD symptoms might vary, gait is one of the motor features of this disorder that is most commonly impaired. Small, shuffling steps, general slowness of movement (hypokinesia), and in severe cases, complete immobility (akinesia), are the characteristic of Parkinsonian gait [93,94,95]. In contrast to longer double supports, PD patients had shorter strides, slower walking speeds during spontaneous ambulation, and higher cyclic rates [96,97,98,99].

Another symptom of PD is a speech disorder. PD patients may stumble over their words, whisper, or falter toward the end of sentences. Whereas most people speak slowly, others do so quickly and even stutter or stammer. Speech issues may be intensified by PD motor symptoms such as less facial expression, slowness, and hunched posture. Deep brain stimulation, surgery, speech therapy, pharmaceutical intervention, and vocal fold augmentation are a few of the therapeutic approaches. Speech therapy for PD patients should be provided as part of a multidisciplinary approach to patient care, despite the fact that managing Parkinsonian dysarthria is clinically difficult [100].

Another feature of PD is small, cramped handwriting called micrographia, which is typically one of the early signs. Micrographia is a neurological condition that results in words that are often small and clustered together as well as other movement symptoms of the disorder. Additionally, symptoms including rigidity, tremor, and slowness of movement can all make it more difficult to write [101].

Recent research has shown that MRI can be used to detect and diagnose PD far sooner than conventional techniques. In order to detect PD, MRIs scan the brain for particular markers. These signs are frequently present even before Parkinson’s symptoms appear [102].

### Medical Approaches for Parkinson’s Disease Diagnosis

Most cases of PD are identified based on their clinical symptoms. An X-ray or blood test cannot verify the disease. Nevertheless, non-invasive diagnostic imaging, such as positron emission tomography (PET), can help a surgeon make a diagnosis. Conventional methods for diagnosing Parkinsonism include the presence of two or more primary symptoms, the absence of additional neurological symptoms upon examination, the absence of a history of additional potential causes, such as the use of anesthetic drugs, head trauma, or stroke, and responsiveness to levodopa or other Parkinson’s medications [66]. Following are some clinical methods that are used to diagnose PD:

a. Medical Treatment

In most cases, medication is used to treat Parkinson’s patients in order to reduce their disease symptoms. Levodopa drugs or anticholinergic pharmaceuticals stimulate the residual substantia nigra cells to create further dopamine whereas levodopa medications suppress part of the acetylcholine production, which restores the homeostasis of the brain’s chemical production. There are a wide variety of side effects associated with each medication class [103]. Levodopa, which was created more than four decades ago, is frequently referred to as the standard of Parkinson’s treatment. Levodopa is used in lower doses in order to reduce the symptoms. This development significantly lessens acute vomiting and nausea that are frequently encountered as levodopa side effects. Levodopa often lessens the tremor, stiffness, and slowness symptoms in individuals. Patients with a lack of spontaneous movement and muscular stiffness benefit the most from it [104].

b. Dopamine Agonists

The brain’s chemical messengers are imitated by drugs like bromocriptine, pergolide, pramipexole, and ropinirole, which cause neurons to respond as they would to dopamine. Medications can be prescribed either alone or in combination with levodopa, and they can be given to patients in the early stages of the illness or to extend the time that levodopa will be effective. Before recommending dopamine agonists to patients, surgeons take into account the fact that these drugs often have greater adverse effects than levodopa [103].

c. COMT Inhibitors

Inhibitors of catechol-O-methyl transferase (COMT) are among the amino groups that contribute to the stability of levodopa levels. Entacapone, tolcapone, and opi-Capone are the three main COMT inhibitors. These medications work by inhibiting the COMT enzyme, which raises blood levels of levodopa without causing it to be peripherally degraded into 3-O-methyldopa (3-OMD) [105]. Dyskinesia and diarrhea may include possible side effects [103].

d. Selegiline

Monoamine oxidase B (MAO-B) is selectively inhibited by the drug selegiline. The major enzyme responsible for the catabolism of dopamine is MAOs, which are intracellular enzymes found on the mitochondrial membrane. It has been established that selegiline may be used safely in PD patients due to its ability to alleviate symptoms when taken as monotherapy, postpone the onset of levodopa, enhance wearing-off in individuals with motor irregularities, and perhaps even have neuroprotective effects. Selegiline may be considered an ancient medication, whereas rasagiline, a more contemporary MAO-B inhibitor, is equivalent in terms of effectiveness [106].

e. Anticholinergic medications

The function of the neurotransmitter acetylcholine (ACh) in the central and autonomic nervous systems is blocked by anticholinergic medicines, which leads to a wide range of both beneficial and undesirable consequences. Since many of the most often given medications for seniors are indicated for problems common to the aged, one-third to one-half of these medications contain anticholinergic effects [107]. These medications are particularly effective in treating tremors, stiffness of the muscles, and antidepressants for Parkinsonism. Due to difficulties and major adverse effects, they are typically not advised for prolonged use in elderly individuals [103].

f. Amantadine

Levodopa-related dyskinesia is typically treated with amantadine as an add-on medication, although more recently, novel long-acting amantadine formulations have been created with additional indications to treat motor fluctuations. Amantadine is hardly associated to impulse control problems and has not been found to produce dyskinesia [108]. Levodopa or anticholinergic medicine may occasionally be used with amantadine. Some of its adverse effects include confusion, sleeplessness, nightmares, irritability, and hallucinations. It may also cause leg swelling [103].

## 5. State of the Art

In order to distinguish PD cases from healthy controls, a variety of modern ML algorithms, including SVM, ANN, logistic regression, naïve Bayes, etc., were successfully used. In this study, numerous databases, including Web of Science, Elsevier, MDPI, Scopus, Science Direct, IEEE Xplore, Springer, and Google Scholar, were utilized to survey relevant papers on PD.

### 5.1. Literature Review Based on Speech, Gait, and Handwriting Patterns to Diagnose PD

E. Avuçlu et al. [109] proposed a method to detect PD using multiple classifiers. In their study, the authors used 195 sound samples and 22 acoustic vocal characteristics in a variety of 75% training and 25% of test data. The ML classifiers used to detect PD are naive Bayes, random forest, SVM, and KNN. According to research, the SVM accurately diagnoses PD patients with a test data accuracy of 67.27% and a training data accuracy of 87.06%. In a survey by KarimiRouzbahani, H et al. [110], the authors used KNN, SVM, and discrimination-based-function (DBF) classifiers for the diagnosis of PD. In their study, they used several parameters like jitter, fundamental frequency, pitch, shimmer, and other statistical measures. The best accuracy among these classifiers was obtained from KNN with a 93.83% accuracy rate and it also provides good performance in other parameters like sensitivity, specificity, and error rate also.

The authors Khamparia, A. et al. [111] used a CNN classifier applied to speech classification datasets. The accuracy reached throughout the training phase, which is over 77%, makes the results optimistic. In accordance with the works mentioned above, A. Bourouhou et al. [112] examined a variety of classifiers to identify individuals who were likely to have PD. They used 40 participants for their investigation, including 20 subjective patients and 20 NC. According to the experimental findings, the naive Bayes classifier has a detection accuracy, sensitivity rate, and specificity rate are 65%, 68%, and 66.6%, respectively. Sharma, A. et al. [113] used three types of classifiers based on KNN, SVM, and multilayer perceptron (MLP) to diagnose PD. Among all these ML classifiers, SVM using an RBF kernel outperformed with an overall classification accuracy rate of 85.294% percent.

A summary of the most recent DL methods for audio signal processing is given in another work by Purwins, H. et al. [114]. The works that have been examined include CNN as well as other long short-term memory (LSTM) architecture models and audio-specific neural network models. Similar to the previous studies, L. Zhang, Y. Qu, et al. [115] detected PD using naive Bayes and other ML approaches. In their method, relevant features were extracted from the voice signal of PD patients and healthy control (HC) subjects using signal processing techniques. The naive Bayes algorithm shows a 69.24% detection accuracy and 96.02% precision rate for the 22 voice characteristics. S. R. Kadiri et al. [116] suggested a technique for detecting PD using SVM on shifted delta cepstral (SDC) and single frequency filtering cepstral coefficients (SFFCC) features extracted from speech signals of PD patients and HC. Comparing the standard MFCC + SDC features with the SDC + SFFCC features, performance increases of 9% were observed. A 73.33% detection accuracy with a 73.32% F1-score is displayed by the conventional SVM on SDC + SFCC features. In addition to the naive Bayes classifier, several additional supervised methods, including but not restricted to well-known DL methods, have been suggested to identify PD patients among healthy controls.

In a survey conducted by M. Pramanik et al. [117], the authors examined two recognizing decision forests, i.e., SysFor and ForestPA, along with the most widely used random forest classifier, which has been utilized as a Parkinson’s detector. In their study, as compared with SysFor and ForestPA, random forest’s average detection accuracy on incremental trees shows 93.58%. For the purpose of classifying PD through sets of acoustic vocal (voice) characteristics, Gunduz, H. [118] suggested two frameworks based on CNN. Both frameworks are used for the mixing of different feature sets, although they combine feature sets in different ways. Although the second framework provides feature sets to the parallel input levels that are directly connected to convolution layers, the first framework first combines several feature sets before passing them as inputs to the nine-layered CNN.

One of the most important technological advancements of the twenty-first century has been the use of AI and ML in society. Basic AI systems were in use in the late 20th century, but during the past ten years, the creation of procedures and systems that employ ML and other functions has risen tremendously. These tools help researchers in a huge range of areas manage their data and work more efficiently. Clinical insights continue to employ AI and ML in a variety of ways. The fact that AI technologies don’t only focus on one component of clinical findings is one of their strongest features. For handling massive and heterogeneous data sources, spotting complicated and hidden patterns, and forecasting difficult outcomes, many ML algorithms are available. Because of this, ML has much to offer in terms of clinical insights across the board, from preclinical drug development to pre-trial planning to study execution to data storage and analysis [119].

AI is assisting physicians in better diagnosing and treating diseases like postoperative hypotension, and more advanced future models may have even more widespread medical uses. The evolutionary step in the creation of therapeutic pathways and adherence is ML. The real benefit of ML, however, is that it enables provider organizations to use information about the patient population from their own systems of record to create therapeutic pathways that are unique to their procedures, clientele, and physicians [120].

However, various algorithms such as SVM, ANN, naive Bayes, ensemble-based method, and gradient-boosted trees [121,122,123,124,125,126] were used to diagnose PD based on speech features where the highest accuracy of 94.93% was obtained from ANN [122]. For the detection of PD using handwriting patterns, several algorithms such as SVM, random forest, and CNN [127,128,129,130,131,132,133] were used where the highest accuracy of 97.23% was obtained from CNN [132]. Similarly, diagnosing PD based on gait parameters using a different algorithm such as SVM, fuzzy KNN, ANN, and deep CNN [134,135,136,137,138,139,140] where the highest accuracy of 100% was obtained from SVM [137].

It can be seen from the reviews above that all the research has been conductedand is only restricted to a small number of datasets. The above previous works inspired us to try a new methodology. In this study, we experimented with several feature selection methods before comparing the results with various ML classifiers. Table 3 represents the review of AI/ML/DL techniques used to diagnose major symptoms of PD i.e., speech recording, handwriting pattern, and gait features for 20 studies.

### 5.2. Literature Review on Neuroimagingto Diagnose PD

Since neuroimaging has demonstrated its efficacy in the diagnosis of PD, CAD that is based on neuroimaging has received a lot of attention. The classifier module is one of the important components of a CAD system that directly affects classification performance [141].

Chakraborty, S. et al. [142] discussed that a total of 906 people participated in the survey, of whom 203 served as controls, 66 as prodromal subjects, and 637 as symptoms of PD. Eight subcortical regions were separated from the obtained MRI scans by using atlas-based segmentation in order to examine the MRI scans for the diagnosis of the subjective disease. In addition, morphological, textural, and statistical information were recovered from the eight extracted subcortical structures using feature extraction. For each MRI scan, an exhaustive collection of 107 features was produced after the feature extraction procedure. In order to determine the optimal feature set for the identification of PD, a two-level feature extraction technique was used.

Bhan et al. [143] proposed a deep-learning methodology to diagnose PD among healthy controls and subjective disease. According to a study, taking the right actions early greatly improves the likelihood of healing, and using a machine to carry out the detection process might save a lot of time. The MRI data of PD participants were effectively separated from healthy controls using the CNN and the LeNet-5 architecture.

A methodology was suggested by Kumar, R. et al. [144] to use a discrete wavelet transform-based fusion of MRI sequences and radiomics feature extraction as the approach for a novel framework for classifying brain tumors. The performance evaluation of the authors’ method was conducted using the Brain Tumor Segmentation 2018 Challenge training dataset, and features were taken from three areas of interest created by combining several tumor regions. The authors employed various ML classifiers to train the model. They also used filter and wrapper method-based feature selection strategies to choose a meaningful collection of features.

In a survey conducted by Pang, Y. et al. [145], the authors assessed a hand and finger motion capture wearable device that is basically used to record the hand and finger motion of HC and subjective disease patients. Using the DWT, the specific three-dimensional motion properties of each finger joint were recovered. By examining the motion variations in the frequency domain on four types of motion from 5 subjective patients and 22 healthy control subjects, the degree of tremor for each finger joint was measured.

According to A. Radziunas, et al. [146], the authors examined 28 PD patients for sleep problems using the PDSS and underwent brain MRIs conducted on 14 males and 14 females, all of whom were between the ages of 58. Using the FreeSurfer program, automated vowel-based image analysis was carried out.

D. Zhang, J. et al. [147] used multivariate pattern analysis to distinguish between subjective patients and NC by using the characteristics of the inconsistency of tremor using a multiple linear regression model. For this experiment, the data were collected from the 36 participants where 16 were affected by PD and 20 matched healthy controls. For each person, wavelet-based functional and morphological brain networks were then built. According to graph-based network analysis, individuals with PD had a disruption in information translation efficiency within the wavelet scale 2 of the functional brain network.

In the study by Kiryu, S. et al. [148], the authors performed the accuracies of diagnostic performances for progressive supranuclear palsy (PSP), multiple system atrophy with predominant parkinsonian features (MSA-P), PD, and HC subjects were 93.7%, 95.2%, 96.8%, and 98.4%, respectively. For separating each disorder from others PSP, MSA-P, PD, and healthy individuals were 98.2%, 99%, 99.5%, and 100%, respectively.

Magesh, P. R. et al. [149] suggested a CNN-based regression approach for differentiating between subjective patients and NC. Data for 252 patients were obtained for this study from the PPMI database. The trained network was tested using ten-fold cross-validation, and the performance parameter was the absolute difference between predicted and actual scores. Evaluation of prediction using inputs with and without DAT images.

Mabrouk, R. et al. [150] proposed five models of ML for distinguishing PD patients and HC using clinical evaluation and image-based features applied later on in the SWEDD group as a potential application of motor and non-motor data in understanding PD characteristics. In binary classification, the five models had a high degree of accuracy (75.4–78.4% for motor characteristics and 71–82.2% for non-motor data). In this manner, the authors have shown how ML models may be used to binary classify SPECT data, proving their applicability and utility.

The study proposed by Quan, J. et al. [151], demonstrates a deep-CNN methodology and assesses the effectiveness of the method for categorizing DaTSCAN SPECT images. The InceptionV3 architecture, which placed second in the 2015 ImageNet Large Scale Visual Recognition Competition (ILSVRC), serves as the foundation model for the deep neural network used in this study. On top of this basis, a unique, binary classifier block was created. The effectiveness of the model was assessed using ten-fold cross-validation in order to adjust for the short dataset size.

In accordance with the aforementioned studies, the authors Moon, S. et al. [152] proposed a number of ML methods, including an SVM, decision tree, gradient boosting, and neural network for the diagnosis of PD patients using an F1-score dummy model. For this study, authors used balance and gait variables collected during the instrumented stand and walk test from people with 524 PD patients and 43 essential tremors (ET).

In the study by Adams, M. P. et al. [153], the authors created a method based on CNN that predicts clinical motor function evaluation scores from longitudinal DAT SPECT images and clinical measurements that are not imaging-based.

In line with the above works, Khachnaoui, H. et al. [154] suggested an ML methodology used to differentiate PD patients from HC within a SWEDD group. The authors analyzed data from 548 participants using principal component analysis (PCA) and linear discriminant analysis (LDA) methods. The authors developed density-based spatial (DBSCAN), K-means, and hierarchical clustering using the results of the best reduction approach. In terms of accuracy, sensitivity, and specificity, hierarchical clustering outperformed DBSCAN and K-means algorithms by 64%, 78.13%, and 38.89%, respectively. The suggested approach showed that ML models could successfully separate PD patients from HC participants within a SWEDD group.

As stated by Oliveira, F. P. et al. [155], the authors aimed to evaluate the possibility of a collection of features derived from FP-CIT SPECT brain images to be employed in computer-aided “in vivo” confirmation of dopaminergic degradation and afterward to support clinical decision-making to diagnose Parkinson’s disease.

According to Saponaro, S et al. [156], the authors addressed the case-control ML separation capacity in the analysis of a multi-center MRI dataset, the authors showed how the use of a harmonization strategy on brain structural variables unlocks this capability. On the ABIDE data collection, which included people across a wide age range, this impact is proven. Following data harmonization, the overall capacity of a random forest classifier to distinguish between autism spectrum disorders (ASD) and normal development (ND) increases from very poor performance (AUC = 0.58 ± 0.04) to a still low but reassuringly significant AUC = 0.67 ± 0.03. AUC = 0.62 ± 0.02, AUC = 0.65 ± 0.03, and AUC = 0.69 ± 0.06, respectively, were obtained when the RF classifier’s performances were assessed in the age-specific subgroups of children, adolescents, and adults.

According to Tufail, A. B. et al. [157], using PET and SPECT neuroimaging modalities to separate Alzheimer’s disease (AD), PD, and NC classes, the authors employed a 3D CNN to extract features for multiclass classification of both AD and PD. Along with random weak Gaussian blurring, random zooming in and out, and discrete cosine transform, both frequency and spatial domain learning techniques have been used.

In the study by Antikainen, E. et al. [158], the authors investigated 23 SPECT image characteristics on 646 individuals for the early detection of PD. The authors demonstrated that matching accuracy may be reached with only eight features, including unique features, and achieve 94% balanced classification accuracy in independent test data utilizing the whole feature space. All of the qualities that are being provided can be produced by commonly accessible clinical software, making it simple to extract and use them.

In the work by Salvatore, C. et al. [159], the authors proposed Morphological T1-weighted MRIs of PD patients (28), PSP patients (28), and HC subjects (28) were used by a supervised machine learning algorithm based on the combination of PCA as feature extraction technique and on SVM as classification algorithm. The algorithm was able to obtain voxel-based morphological biomarkers of PD and PSP.

The authors Martínez-Ibañez, M., et al. [160] discussed computing isosurfaces as a method of removing pertinent information from 3D brain images. These isosurfaces are then used to implement a computer-aided diagnosis (CAD) system to help with the diagnosis of PD. This system uses the most well-known CNN architecture, LeNet, to classify DaTSCAN images with an average accuracy of 95.1% and AUC = 97%, obtaining comparable (slightly better) values to those obtained for the majority of the recently proposed systems. Therefore, it may be inferred that computing isosurfaces considerably decrease the complexity of the inputs, producing good classification accuracy with little processing load.

According to the above-mentioned work, Kurmi, A. et al. [161] suggested utilizing DaTSCAN images to predict Parkinson’s using a collection of DL models. The classification of PD was initially conducted using four DL models: VGG16, ResNet50, Inception-V3, and Xception. To improve the classification model’s overall performance, they used a Fuzzy Fusion logic-based ensemble technique in the next step. The suggested model outperforms the individual model in terms of attained recognition accuracy, precision, sensitivity, specificity, and F1-score, which are each 98.45%, 98.84%, 98.84%, 97.67%, and 98.84%, respectively. Additionally, they have created a software application with a graphical user interface (GUI) for the general public that accurately and promptly identifies all classes in MRI.

The evaluations mentioned above demonstrate that all the research has been completed and is only limited to a few datasets. The aforementioned earlier efforts motivated us to take a different approach. In this work, we evaluated a variety of feature selection techniques and then compared the outcomes with a number of ML classifiers. Tremor, DaTSCAN, SPECT, and MRI-T are some of the key symptoms of PD that may be diagnosed with ML methods, as shown in Table 4.

## 6. Discussion: Challenges and Recommendations

Although there is no known cure for PD, with an accurate and timely diagnosis, we can reduce and control its progression. Compared with traditional PD detection methods, AI is a strong choice for detecting early-stage PD. The use of AI can help with global epidemiology initiatives and patient symptom monitoring. Despite how stimulating these applications are, it is important to consider both the value and potential limitations of these cutting-edge analytical techniques. The most promising applications of AI are yet futuristic [180,181,182].

In this section, we summarized the current limitations and challenges and made prospective suggestions (recommendations) for the future that might lead to efficient AI and ML methods to address the issues.

### 6.1. Current Limitations and Challenges

Currently, DL-based CAD systems are usually applied as diagnostic aids or for educational purposes [183]. With the help of useful research, the software may now be developed in the real world to diagnose Parkinson’s disease utilizing MRI modalities. However, there are still a few challenges faced by researchers which are listed as follows:

a. Issues with Multimodality datasets for PD detection

Another difficulty in diagnosing PD for researchers is the unavailability of multimodality neuroimaging datasets. The diagnosis of brain disorders including PD, AD, and schizophrenia (SZ) is often greatly aided by multimodality neuroimaging data [184]. Various clinical investigations have described the reliable detection of PD using a combination of neuroimaging modalities, such as EEG-fMRI [185,186,187,188], MRI-PET [189,190,191,192], fMRI-MEG [193,194], and fMRI-sMRI [195,196,197]. Diagnosis of PD using multimodality neuroimaging data is complicated and time-consuming for doctors, despite all the advantages. The lack of multimodality neuroimaging datasets for PD detection has been a significant problem for researchers. The accessibility of multimodality neuroimaging datasets might result in significant research on PD diagnosis utilizing AI methods.

b. Issues with Machine Learning Techniques

Another challenge to diagnosing PD is related to the use of ML techniques, including that the most essential aspect of CADS is extracting the distinctive features that might result in useful PD biomarkers. An extensive understanding of the AI area is needed to implement CADS based on ML. It is challenging to choose the algorithms for each component of an ML-based CADS in order to make a highly precise PD diagnosis. ML-based CADS, however, is not a function that should be used for large amounts of data input. The fact that MRI uses several imaging procedures and does not have a function that is appropriate for processing these data simultaneously presents another challenge. Developing practical software for detecting Parkinson’s disease is quite challenging due to these challenges.

c. Issues with Clinical Validation

The effectiveness of deep learning models for PD detection is mostly evaluated using standard ML parameters including accuracy, sensitivity, specificity, and/or area under the curve of a receiver operating characteristic curve. These measures may not accurately represent clinical effectiveness and anticipated positive adjustments to patient treatment. Additionally, before using AI-powered diagnostic tools in the clinic, physicians must receive training on how to use them because some of these metrics are difficult to interpret [198]. To support the validity of the suggested DL framework for PD diagnosis, recent studies [199,200,201,202] addressing this problem performed a connection of model predictions with neuropathological results as well as a head-to-head evaluation of the system performance with a team of neurologists. Due to data inconsistencies, it is important to do clinical validation to make sure that the imaging-specific features are appropriately compatible with the intended clinical adoption. A CAD system must be modified for a new community through clinical adoption [160], which requires certain members of the deployment population’s intended target demographic.

### 6.2. Recommendations and Future Perspectives

We examined both the benefits and disadvantages of the selected articles. After taking into account the recommendations for critical evaluations [203], we began searching for relevant directions for further research. We divided our findings into groups that address the same or related problems and defined them as follows:

- Make the most of all available data sources. It might be difficult to gather all available modalities for each subject. For instance, a PET scan, a costly neuroimaging modality, is not performed on some participants, although practically all of the subjects’ clinical records list an MRI. This is true for publicly accessible data like ADNI. To fill up the gaps in the data, we advise employing certain methods. For instance, utilizing MRI data to complete missing PET data [204], creating CT scans from MRIs [205,206,207], cycle-consistent generative adversarial networks (GAN) [208], and feature-consistent GAN [207]. As an alternative, deep designs that include handling procedures for missing data can be applied [209,210,211,212,213]. Additionally, data augmentation might be useful in this context to increase the dataset and address unbalanced classes. Through image modification operators including rotation [214], scaling and shifting [215], changing intensity, contrast, and saturation [216], as well as noise injection and random translation [217], data augmentation may be accomplished.
- The widespread usage of the CNN algorithm [218,219] on MRI image data is a significant discovery. Comparing these models to other algorithms, they frequently produce favorable outcomes. Researchers might wish to conduct further research and use more CNN-based hybrid algorithms [220,221,222]. Additionally, we found that classifying images has not frequently utilized the learning algorithm. This is an opportunity for future researchers to utilize attention to raise the precision of deep learning models.
- At present, wearable sensors are only useful for using gait parameters to diagnose PD. The wearable device that can detect PD must have the other modules included in it. In addition to one symptom, researchers must concentrate on creating wearable sensor systems that can diagnose additional symptoms also. For instance, a wrist-worn sensor might be created that can track data constantly over a long period of time and recognize various PD symptoms.
- Currently, various ML models have been developed by researchers that can diagnose PD based on a patient’s specific symptoms. The researchers should focus on establishing an ML model for diagnosing PD that takes all the symptoms as input. A lightweight portable device can be used to diagnose the various symptoms of PD by measuring several parameters such as accuracy, precision, sensitivity, recall, etc. This device should be easily wearable and washable, and it should be able to identify the different stages of the disease, along with analyzing the changes due to medication treatment.

## 7. Conclusions

AI and ML are revolutionizing healthcare since technologies assist in the diagnosis of any disease and have made it easier in recent years. This technique has the potential to revolutionize healthcare with more accuracy in diagnosing a disease. A computerized system aids doctors in making more precise diagnoses, forecasting patients’ future health, and making better treatment recommendations. In this work, we conducted a comprehensive review of 217 research papers that addressed the application of various machine learning methods and deep neural network architectures to diagnose PD. We also thoroughly examined and analyzed the researcher’s architectural designs. This review is significant for the advancements in neural networks and associated learning systems, which offer insightful information and recommendations for future growth.

## Figures and Tables

**Table 1:** Major influential journals publishing on diagnosis of PD (2015–2022) from Web of Science database.

Journal Name	No. of Articles	Publisher	Indexing
Sensors	47	MDPI	SCIE and Scopus
IEEE Access	32	IEEE	SCIE
Plos One	23	Public Library Science	SCIE
Frontiers in Neurology	17	Frontiers Media Sa	SCIE
Frontiers in Neuroscience	14	Frontiers Media Sa	SCIE
IEEE journal of biomedical and health informatics	14	IEEE	SCIE
Diagnostics	13	MDPI	SCIE and Scopus
Applied Sciences Basel	11	MDPI	SCIE and Scopus
IEEE Transactions on Neural Systems and Rehabilitation Engineering	11	IEEE	SCIE
IEEE Sensors Journal	10	IEEE	SCIE
NPJ Parkinson’s Disease	9	Nature	SCIE
Brain Sciences	8	MDPI	SCIE and Scopus
IEEE Transactions on Biomedical Engineering	7	IEEE	SCIE
Multimedia Tools and Applications	6	Springer	SCIE
Journal of Healthcare Engineering	5	Hindawi	SCIE
IEEE Journal of Transactional Engineering in Health and Medicine	4	IEEE	SCIE
Nature Communications	3	Nature Portfolio	SCIE
Applied Acoustics	2	Elsevier	SCIE
Electronics	2	MDPI	SCIE and Scopus
IEEE ACM Transactions on Audio Speech and Language Processing	2	IEEE	SCIE
IEEE Transactions on Automation Science And Engineering	2	IEEE	SCIE
IEEE Transactions on Biomedical Circuits and Systems	2	IEEE	SCIE
IEEE Transactions on Radiations and Plasma Medical Sciences	2	IEEE	SCIE

**Table 2:** Summary of review article to diagnose PD (2015–2022) from Web of Science database.

Authors	Scope of the Review	Citations	Type of Study
Sibley, KG et al., 2021 [41]	Analysis of Parkinson’s disease severity based on videos	16	A brief review
Belic, M et al., 2019 [42]	Using artificial intelligence to aid in the diagnosis and evaluation of Parkinson’s disease	74	A review
Landers, M et al., 2021 [43]	Can artificial intelligence diagnose and treat Parkinson’s disease instead of a movement disorders specialist?	1	A review
Palumbo, B et al., 2021 [44]	In order to more accurately diagnose Parkinson’s disease and Parkinsonian symptoms, artificial intelligence approaches enhance nuclear medicine modalities.	3	A review
Saravanan, S et al., 2022 [45]	Artificial intelligence (AI)-based approaches for the diagnosis of Parkinson’s disease.	1	A systematic review
Perju-Dumbrava, L et al., 2022 [46]	Applications of robotic technology and artificial intelligence in Parkinson’s disease.	0	A review
Giannakopoulou, KM et al., 2022 [47]	Methods of the Iot technology and machine learning for the detection, monitoring, and management of Parkinson’s disease.	2	A systematic review
Khachnaoui, H et al., 2020 [48]	PET/SPECT imaging for Parkinson’s disease using machine learning and deep learning.	5	A review
Narayanan, RR et al., 2022 [49]	The effects of artificial intelligence (AI) on drug discovery and product development.	0	A review
Termine, A et al., 2021 [50]	A multi-layer view of neurodegenerative diseases: insights from the application of artificial intelligence to big data.	8	A review
Lim, ACY et al., 2022 [51]	Adult gait analysis and the diagnosis of disorders that modify their stride using artificial intelligence and personalised algorithms with inertial wearable technology.	1	A review
Xu, JJ et al., 2019 [52]	Parkinson’s disease diagnosis studies using magnetic resonance imaging and artificial intelligence.	21	A review
Zhang, Z et al., 2021 [53]	Artificial intelligence used to classify human brain neurological and psychiatric disorders using MRI.	3	A scoping review
Yadav, D et al., 2020 [54]	Intelligent diagnostic tools using mechanobiological and artificial intelligence methods.	2	A review
Patil, AD et al., 2022 [55]	An understanding of neurodegenerative disease with artificial intelligence in ophthalmology.	0	A review
Suri, JS et al., 2022 [56]	Using the atherosclerosis pathway and an artificial intelligence paradigm, cardiovascular/stroke risk stratification in Parkinson’s disease patients.	7	A systematic review
Vitale, A et al., 2021 [57]	Neuroimaging data from Parkinson’s symptoms using artificial intelligence.	3	A review
Cascianelli, S et al., 2017 [58]	Molecular imaging modalities in neurodegenerative diseases.	21	A review
Rana, A et al., 2022 [59]	Detection of Parkinson’s disease: the critical role of machine learning algorithms.	0	A review
Raghavendra, U et al., 2022 [60]	Automated diagnosis of neurological disorders using artificial intelligence techniques.	81	A review
Singh, AV et al., 2021 [61]	Artificial intelligence and nanorobotics: anew approach to cross the BBB.	31	A review
Maitin, AM et al., 2022 [62]	Analysis of EEG signals for Parkinson’s disease using machine learning techniques.	1	A systematic review
Vatansever, S et al., 2021 [63]	Using artificial intelligence and machine learning to help in medication development for illnesses of the central nervous system.	38	State–of–the–art
Hansen, C et al., 2018 [64]	How electronic health records and mobile health technology will change Parkinson’s disease patient care.	29	A review
Luis-Martinez, R et al., 2020 [65]	Using digital technology to integrate multidisciplinary care for Parkinson’s disease.	21	A review
Fiandaca, MS et al., 2020 [66]	Advances in Parkinson’s disease and other neurological diseases gene treatments, approaches, and technology.	12	A review
Kubota, KJ et al., 2016 [67]	Large-scale wearable sensor data for Parkinson’s disease using machine learning.	180	A review

**Table 3:** Comparative research on Parkinson’s disease diagnosis using machine learning approaches (a. speech, b. handwriting patterns, and c. gait parameter).

**a.** **Speech Parameter**							
**Reference**	**Modality**	**Algorithms Used**	**Objective**	**Tools**	**Source of Data**	**Subjects**	**Performance**
Sakar et al., 2019 [121]	Speech	Support Vector Machine	Classification of PD from HC	JupyterLab with python programming language	Collected from participants	188 PD and 64 HC	Accuracy (ACC.)—86%
Yasar A. et al., 2019 [122]	Speech	Artificial Neural Network	Classification of PD from HC	MATLAB	Collected from participants	40 PD and 40 HC	ACC.—94.93%
Ouhmida, A, 2021 [123]	Speech	SVM, K-NN, Decision Tree	Classification of PD from HC	Not mentioned	UCI machine learning repository	Not mentioned	AUC-98.26%
Marar et al., 2018 [124]	Speech	Naive Bayes	Classification of PD from HC	R programming	Collected from participants	23 PD and 8 HC	ACC.—94.87%
Sheibani R etal., 2019 [125]	Speech	Ensemble-Based Method	Classification of PD from HC	Python programming	UCI machine learning repository	23 PD and 8 HC	ACC.—90.6%
John M. Tracy etal., 2020 [126]	Speech	Gradient Boosted Trees	Classification of PD from HC	Python	Not mentioned	246 PD and 2023 HC	ACC.—79.7%
**b.** **Handwriting Patterns**							
**Reference**	**Modality**	**Algorithms Used**	**Objective**	**Tools**	**Source of Data**	**Subjects**	**Performance**
Cibulka et al., 2019 [127]	Handwriting Patterns	Random Forest	Classification of PD from HC	Not mentioned	Collected from participants	150 PD and 120 HC	Not mentioned
Hsu S-Y et al., 2019 [128]	Handwriting Patterns	Support Vector Machine	Classification of PD from HC	Weka	PACS	196 PD and 6 HC	ACC.—83.2%
Drotár, P et al., 2016 [129]	Handwriting Patterns	K-NN, Ensemble AdaBoostClassifier, Support Vector Machine	Classification of PD from HC	Python [scikit-learn library]	PaHaW database	37 PD and 38 HC	ACC.—81.3%
Fabian Maass etal., 2020 [130]	Handwriting Patterns	Support Vector Machine	Classification of PD from HC	Not mentioned	Collected from participants	82 PD and 68 HC	Sensitivity—80%
J. Mucha et al., 2018 [131]	Handwriting Patterns	Random Forest Classifier	Classification of PD from HC	Not mentioned	Collected from participants	33 PD and 36 HC	ACC.—90% and Sensitivity—89%
Wenzel et al., 2019 [132]	Handwriting Patterns	Convolutional Neural Network	Classification of PD from HC	MATLAB	Not mentioned	438 PD and 207 HC	ACC.—97.23%
Segovia, F. etal., 2019 [133]	Handwriting Patterns	Support Vector Machine	Classification of PD from HC	Python programming	Not mentioned	95 PD and 94 HC	ACC.—94.2%
**c.** **Gait Parameter**							
**Reference**	**Modality**	**Algorithms Used**	**Objective**	**Tools**	**Source of Data**	**Subjects**	**Performance**
Ye, Q. et al., 2018 [134]	Gait	Support Vector Machine	Classification of PD from HC	Not mentioned	Collected from participants	15 PD and 16 HC	ACC.—90.32%
Klomsae, A et al., 2018 [135]	Gait	Fuzzy KNN	Classification of PD from HC	Not mentioned	Collected from participants	15 PD and 16 HC	ACC.—96.43%
J. P. Félix et al., 2019 [136]	Gait	Support Vector Machine	Classification of PD from HC	MATLAB	Not mentioned	15 PD and 16 HC	ACC.—96.8%
Andrei et al., 2019 [137]	Gait	SVM	Classification of PD from HC	Not mentioned	Laboratory for Gait & Neurodynamics	93 PD and 73 HC	ACC.—100%
Priya SJ et al., 2021 [138]	Gait	ANN	Classification of PD from HC	MATLAB	Laboratory for Gait & Neurodynamics	93 PD and 73 HC	ACC.—96.28%
Oğul, et al., 2020 [139]	Gait	ANN	Classification of PD from HC	MATLAB	Laboratory for Gait & Neurodynamics	93 PD and 73 HC	ACC.—98.3%
Li B et al., 2020 [140]	Gait	Deep CNN	Classification of PD from HC	Not mentioned	Collected from participants	10 PD and 10 HC	ACC.—91.9%

**Table 4:** Comparative research on Parkinson’s disease diagnosis using machine learning approaches (neuroimaging-based).

Reference	Modality	Algorithms Used	Objective	Tools	Source of Data	Subjects	Performance
Hosseini and Makki, 2013 [162]	Essential Tremor (ET)	Auto Associative Neural Network	Classification of ET, PD from HC	Not Mentioned	Collected from participants	20 ET and 20 PD and	ACC.—87.5%
Challa et al., 2016 [163]	DaTSCAN SPECT	Boosted Logistic Regression	Classification of PD from HC	Weka	PPMI Database	402 PD	ACC.—97.159%
Choi et al. 2017 [164]	SPECT	Deep Convolutional Neural Network	Classification of PD from HC	MATLAB	PPMI Database	431 PD, 193 HC, 77 SWEDD	ACC.—98.8% and sensitivity—98.6%
Kim, Wit, and Thurston 2018 [165]	SPECT	Inception-V3 (Pre-trained)	Classification of PD from HC	Not mentioned	Not mentioned	54 PD and 54 HC	Sensitivity—96.3%
Esmaeilzadeh et al., 2018 [166]	MRI-T	Convolutional Neural Network	Classification of PD from HC	Not mentioned	PPMI Database	452 PD and 204 HC	ACC.—100%
Kim, Lee, et al., 2018 [167]	Tremor	Convolutional Neural Network	Classification of PD from HC	Not mentioned	Collected from the participants	92 PD and 95 HC	ACC.—85%
Martinez- Murcia et al., 2017 [168]	SPECT	Convolutional Neural Network	Classification of PD from HC	Not mentioned	PPMI Database	158 PD and 32 SWEDD and 111 HC	ACC.-PD vs HC: 95.5 ± 0.44 and sensitivity-PD vs HC: 96.2 ± 0.051
Qin et al., 2019 [169]	Tremor	Convolutional Neural Network	Classification of PD from HC	Not mentioned	Collected from the participants	147 PD	ACC.—90.55%
Kollia et al., 2019 [170]	MRI and DaTSCAN	Convolutional Neural Network and Recurrent Neural Network	Classification of PD from HC	Not mentioned	Not mentioned	55 PD and 23HC	ACC.—98%
Szumilas et al., 2020 [171]	Tremor	Recurrent Neural Network	Develop a prediction model to evaluate tremor severity in PD patients	Not mentioned	Collected from the participants	64 PD	Not mentioned
Oktay and Kocer 2020 [172]	Tremor	Convolutional Long Short-Term Memory	Classification of PD from HC	C++ with Leap motion API	Medical Faculty Teaching Hospital Neurology Istanbul Medeniyet University	23 Parkinson’s tremors and 17 ET	ACC.—90%
Shahtalebi, S et al., 2020 [173]	Tremor	3D Convolutional Neural Network	Develop a deep recurrent model to predict and eliminate the PHT component of hand motion	Not mentioned	Collected from the participants	81 PD	Not mentioned
Veeraragavan et al., 2020 [174]	MRI	Artificial Neural Network	Classification of PD from HC	Not mentioned	Collected from the participants	93 PD and 73 HC	ACC.—97.41% and sensitivity—97.70%
Chien et al., 2021 [175]	DAT-SPECT	Artificial Neural Network	Classification of PD from HC	MATLAB 2018B	Collected from the participants	234 PD	ACC.—99.22% and sensitivity—81.8%
Yasaka et al., 2021 [176]	MRI	2D Convolutional Neural Network	Classification of PD from HC	MATLAB	Collected from the participants from Juntendo University Hospital	115 PD and 115 HC	Not Mentioned
Yang et al., 2021 [177]	MRI + CI	Ensemble (SVM, RF, KNN, ANN, LR)	Classification of PD from HC	Not mentioned	Not mentioned	65 PD and 36 HC	ACC.—96.88% and sensitivity—95.0%
Vyas et al., 2021 [178]	MRI	2D and 3D Convolutional Neural Network	Classification of PD from HC	Not mentioned	PPMI Database	236 PD and 82 HC	ACC. from 2D and 3D 88.9% and 72.22%, respectively, and sensitivity—92% and 100%, respectively
Yadav 2021 [179]	fMRI	Bayesian 3D-Convolutional Neural Network	Classification of PD from HC	Not mentioned	ADNI Dataset	15 PD and 15 HC	ACC.—97.92%

## Abbreviations

The following abbreviations are used in this manuscript:

3-OMD	3-O-Methyldopa
ACC	Accuracy
ACh	Acetylcholine
AD	Alzheimer’s Disease
AI	Artificial Intelligence
ANN	Artificial Neural Network
ASD	Autism Spectrum Disorders
AUC	Area Under the Curve
CAD	Computer-Aided Diagnosis
CNN	Convolutional Neural Network
COMT	Catechol-O-Methyl Transferase
CSF	Cerebrospinal Fluid
DaTSCAN	Dopamine Transporter Scan
DBF	Discrimination-Based Function
DBSCAN	Density-Based Spatial
DL	Deep Learning
DWT	Discrete Wavelet Transform
ET	Essential Tremors
GAN	Generative Adversarial Networks
GD	Gradient Descent
GDS	Geriatric Depression Scale
HC	Healthy Controls
IEEE	Institute of Electrical and Electronics Engineers
KNN	K-Nearest Neighbor
LDA	Linear Discriminant Analysis
LIME	Local Interpretable Model-Agnostic Explainer
LSTM	Long Short-Term Memory
LSVRC	Large Scale Visual Recognition Competition
MAO-B	Monoamine oxidase B
MDPI	Multidisciplinary Digital Publishing Institute
MFCC	Mel-Frequency Cepstral Coefficients
ML	Machine Learning
MLP	Multilayer Perceptron
MoCA	Montreal Cognitive Assessment
MMSE	Mini-Mental Score examination
MSA-P	Multiple System Atrophy with Predominant Parkinsonian Features
MVDA	Multi-Variate Vocal Data Analysis
NB	Naive Bayes
NC	Normal Control
ND	Normal Development
NLP	Natural Language Processing
OCT	Optical Coherence Tomography
PCA	Principal Component Analysis
PD	Parkinson’s Disease
PDSS	Panic Disorder Severity Scale
PET	Positron Emission Tomography
PHT	Pathological Hand Tremor
PIGD	Postural Instability Gait Disorder
PLOS	Public Library of Science
PPMI	Parkinson’s Progression Markers Initiative
PRISMA	Preferred Reporting Items for Systematic Reviews and Meta-Analyses
PSP	Progressive Supranuclear Palsy
RBD	Random Eye Movement Sleep Behavior Disorder
RL	Reinforcement Learning
SCOPA-AUT	Scales for Outcomes in Parkinson’s Disease-Autonomic
SDC	Shifted Delta Cepstral
SFFCC	Single Frequency Filtering Cepstral Coefficients
SL	Supervised Learning
sMRI	Structural Magnetic Resonance Imaging
SPECT	Single-Photon Emission Computerized Tomography
STAI	State-Trait Anxiety Inventory for Adults
SVM	Support Vector Machine
SZ	Schizophrenia
UL	Unsupervised Learning
UPDRS	Unified Parkinson’s Disease Rating Scale
WHO	World Health Organization

## References

[1] DeMaagd G., Philip A. (2015). Parkinson’s Disease and Its Management: Part 1: Disease Entity, Risk Factors, Pathophysiology, Clinical Presentation, and Diagnosis. Pharm. Ther..

[2] Rizek P., Kumar N., Jog M.S. (2016). An update on the diagnosis and treatment of Parkinson disease. CMAJ.

[3] Arias-Vergara T., Vásquez-Correa J.C., Orozco-Arroyave J.R. (2017). Parkinson’s Disease and Aging: Analysis of Their Effect in Phonation and Articulation of Speech. Cogn. Comput..

[4] De Rijk M.D., Launer L.J., Berger K., Breteler M.M., Dartigues J.F., Baldereschi M., Fratiglioni L., Lobo A., Martinez-Lage J., Trenkwalder C. (2000). Prevalence of Parkinson’s disease in Europe: A collaborative study of population-based cohorts. Neurologic Diseases in the Elderly Research Group. Neurology.

[5] Cantürk İ., Karabiber F. (2016). A machine learning system for the diagnosis of Parkinson’s disease from speech signals and its application to multiple speech signal types. Arab. J. Sci. Eng..

[6] Singh N., Pillay V., Choonara Y.E. (2007). Advances in the treatment of Parkinson’s disease. Prog. Neurobiol..

[7] Sakar B.E., Isenkul M.E., Sakar C.O., Sertbas A., Gurgen F., Delil S., Apaydin H., Kursun O. (2013). Collection and analysis of a Parkinson speech dataset with multiple types of sound recordings. IEEE J. Biomed. Health Inform..

[8] Abujrida H., Agu E., Pahlavan K. Smartphone-based gait assessment to infer Parkinson’s disease severity using crowdsourced data. Proceedings of the 2017 IEEE Healthcare Innovations and Point of Care Technologies (HI-POCT).

[9] Adams W.R. (2017). High-accuracy detection of early Parkinson’s Disease using multiple characteristics of finger movement while typing. PLoS ONE.

[10] Adeli E., Shi F., An L., Wee C.-Y., Wu G., Wang T., Shen D. (2016). Joint feature-sample selection and robust diagnosis of Parkinson’s disease from MRI data. Neuroimage.

[11] Adeli E., Thung K.-H., An L., Wu G., Shi F., Wang T., Shen D. (2019). Semi-Supervised Discriminative Classification Robust to Sample-Outliers and Feature-Noises. IEEE Trans. Pattern Anal. Mach. Intell..

[12] Agarwal A., Chandrayan S., Sahu S.S. Prediction of Parkinson’s disease using speech signal with Extreme Learning Machine. Proceedings of the 2016 International Conference on Electrical, Electronics, and Optimization Techniques (ICEEOT).

[13] Ahmadi S.-A., Vivar G., Frei J., Nowoshilow S., Bardins S., Brandt T., Krafczyk S. (2019). Towards computerized diagnosis of neurological stance disorders: Data mining and machine learning of posturography and sway. J. Neurol..

[14] Aich S., Kim H., younga K., Hui K.L., Al-Absi A.A., Sain M. A Supervised Machine Learning Approach Using Different Feature Selection Techniques on Voice Datasets for Prediction of Parkinson’s Disease. Proceedings of the 2019 21st International Conference on Advanced Communication Technology (ICACT).

[15] Al-Fatlawi A.H., Jabardi M.H., Ling S.H. Efficient diagnosis system for Parkinson’s disease using deep belief network. Proceedings of the 2016 IEEE Congress on Evolutionary Computation (CEC).

[16] Alam M.N., Garg A., Munia T.T.K., Fazel-Rezai R., Tavakolian K. (2017). Vertical ground reaction force marker for Parkinson’s disease. PLoS ONE.

[17] Alaskar H., Hussain A. Prediction of Parkinson Disease Using Gait Signals. Proceedings of the 2018 11th International Conference on Developments in eSystems Engineering (DeSE).

[18] Alharthi A.S., Ozanyan K.B. Deep Learning for Ground Reaction Force Data Analysis: Application to Wide-Area Floor Sensing. Proceedings of the 2019 IEEE 28th International Symposium on Industrial Electronics (ISIE).

[19] Ali L., Khan S.U., Arshad M., Ali S., Anwar M. A Multi-model Framework for Evaluating Type of Speech Samples having Complementary Information about Parkinson’s Disease. Proceedings of the 2019 International Conference on Electrical, Communication, and Computer Engineering (ICECCE).

[20] Ali L., Zhu C., Golilarz N.A., Javeed A., Zhou M., Liu Y. (2019). Reliable Parkinson’s Disease Detection by Analyzing Handwritten Drawings: Construction of an Unbiased Cascaded Learning System Based on Feature Selection and Adaptive Boosting Model. IEEE Access.

[21] Ali L., Zhu C., Zhang Z., Liu Y. (2019). Automated Detection of Parkinson’s Disease Based on Multiple Types of Sustained Phonations Using Linear Discriminant Analysis and Genetically Optimized Neural Network. IEEE J. Transl. Eng. Health Med..

[22] Alqahtani E.J., Alshamrani F.H., Syed H.F., Olatunji S.O. Classification of Parkinson’s Disease Using NNge Classification Algorithm. Proceedings of the 2018 21st Saudi Computer Society National Computer Conference (NCC).

[23] Amoroso N., la Rocca M., Monaco A., Bellotti R., Tangaro S. (2018). Complex networks reveal early MRI markers of Parkinson’s disease. Med. Image Anal..

[24] Anand A., Haque M.A., Alex J.S.R., Venkatesan N. Evaluation of Machine learning and Deep learning algorithms combined with dimentionality reduction techniques for classification of Parkinson’s Disease. Proceedings of the 2018 IEEE International Symposium on Signal Processing and Information Technology (ISSPIT).

[25] Khodatars M., Shoeibi A., Sadeghi D., Ghaasemi N., Jafari M., Moridian P., Berk M. (2021). Deep learning for neuroimaging-based diagnosis and rehabilitation of autism spectrum disorder: A review. Comput. Biol. Med..

[26] Baby M.S., Saji A.J., Kumar C.S. Parkinsons disease classification using wavelet transform based feature extraction of gait data. Proceedings of the 2017 International Conference on Circuit, Power and Computing Technologies (ICCPCT).

[27] Baggio H.C., Abos A., Segura B., Campabadal A., Uribe C., Giraldo D.M., Perez-Soriano A., Muñoz E., Compta Y., Junque C. (2019). Cerebellar resting-state functional connectivity in Parkinson’s disease and multiple system atrophy: Characterization of abnormalities and potential for differential diagnosis at the single-patient level. Neuroimage Clin..

[28] Bakar Z.A., Ispawi D.I., Ibrahim N.F., Tahir N.M. Classification of Parkinson’s disease based on Multilayer Perceptrons (MLPs) Neural Network and ANOVA as a feature extraction. Proceedings of the 2012 IEEE 8th International Colloquium on Signal Processing and Its Applications.

[29] Banerjee M., Chakraborty R., Archer D., Vaillancourt D., Vemuri B.C. DMR-CNN: A CNN Tailored for DMR Scans with Applications to PD Classification. Proceedings of the 2019 IEEE 16th International Symposium on Biomedical Imaging (ISBI 2019).

[30] Benba A., Jilbab A., Hammouch A. (2016). Discriminating between Patients with Parkinson’s and Neurological Diseases Using Cepstral Analysis. IEEE Trans. Neural Syst. Rehabil. Eng..

[31] Benba A., Jilbab A., Hammouch A., Sandabad S. Using RASTA-PLP for discriminating between different Neurological diseases. Proceedings of the 2016 International Conference on Electrical and Information Technologies (ICEIT).

[32] Bernad-Elazari H., Herman T., Mirelman A., Gazit E., Giladi N., Hausdorff J.M. (2016). Objective characterization of daily living transitions in patients with Parkinson’s disease using a single body-fixed sensor. J. Neurol..

[33] Bhati S., Velazquez L.M., Villalba J., Dehak N. LSTM Siamese Network for Parkinson’s Disease Detection from Speech. Proceedings of the 2019 IEEE Global Conference on Signal and Information Processing (GlobalSIP).

[34] Buongiorno D., Bortone I., Cascarano G.D., Trotta G.F., Brunetti A., Bevilacqua V. (2019). A low-cost vision system based on the analysis of motor features for recognition and severity rating of Parkinson’s Disease. BMC Med. Inform. Decis. Mak..

[35] Lakany H. (2008). Extracting a diagnostic gait signature. Pattern Recogn..

[36] Figueiredo J., Santos C.P., Moreno J.C. (2018). Automatic recognition of gait patterns in human motor disorders using machine learning: A review. Med. Eng. Phys..

[37] Hazan H., Hilu D., Manevitz L., Ramig L.O., Sapir S. Early diagnosis of Parkinson’s disease via machine learning on speech data. Proceedings of the 27th Convention of Electrical and Electronics Engineers in Israel.

[38] Karan B., Sahu S.S., Mahto K. (2020). Parkinson disease prediction using intrinsic mode function based features from speech signal. Biocybern. Biomed. Eng..

[39] Frid A., Hazan H., Hilu D., Manevitz L., Ramig L.O., Sapir S. Computational diagnosis of Parkinson’s Disease directly from natural speech using machine learning techniques. Proceedings of the International Conference on Software Science, Technology and Engineering.

[40] Page M.J., McKenzie J.E., Bossuyt P.M., Boutron I., Hoffmann T.C., Mulrow C.D., Shamseer L., Tetzlaff J.M., Akl E.A., Brennan S.E. (2021). The PRISMA 2020 Statement: An Updated Guideline for Reporting Systematic Reviews. BMJ.

[41] Sibley K.G., Girges C., Hoque E., Foltynie T. (2021). Video-based analyses of Parkinson’s disease severity: A brief review. J. Parkinson’s Dis..

[42] Belić M., Bobić V., Badža M., Šolaja N., Đurić-Jovičić M., Kostić V.S. (2019). Artificial intelligence for assisting diagnostics and assessment of Parkinson’s disease—A review. Clin. Neurol. Neurosurg..

[43] Landers M., Saria S., Espay A.J. (2021). Will Artificial Intelligence Replace the Movement Disorders Specialist for Diagnosing and Managing Parkinson’s Disease?. J. Parkinson’s Dis..

[44] Palumbo B., Bianconi F., Nuvoli S., Spanu A., Fravolini M.L. (2021). Artificial intelligence techniques support nuclear medicine modalities to improve the diagnosis of Parkinson’s disease and Parkinsonian syndromes. Clin. Transl. Imaging.

[45] Saravanan S., Ramkumar K., Adalarasu K., Sivanandam V., Kumar S.R., Stalin S., Amirtharajan R. (2022). A Systematic Review of Artificial Intelligence (AI) Based Approaches for the Diagnosis of Parkinson’s Disease. Arch. Comput. Methods Eng..

[46] Perju-Dumbrava L., Barsan M., Leucuta D.C., Popa L.C., Pop C., Tohanean N., Popa S.L. (2022). Artificial intelligence applications and robotic systems in Parkinson’s disease. Exp. Ther. Med..

[47] Giannakopoulou K.M., Roussaki I., Demestichas K. (2022). Internet of Things Technologies and Machine Learning Methods for Parkinson’s Disease Diagnosis, Monitoring and Management: A Systematic Review. Sensors.

[48] Khachnaoui H., Mabrouk R., Khlifa N. (2020). Machine learning and deep learning for clinical data and PET/SPECT imaging in Parkinson’s disease: A review. IET Image Process..

[49] Narayanan R.R., Durga N., Nagalakshmi S. (2022). Impact of Artificial Intelligence (AI) on Drug Discovery and Product Development. Indian J. Pharm. Educ. Res..

[50] Termine A., Fabrizio C., Strafella C., Caputo V., Petrosini L., Caltagirone C., Cascella R. (2021). Multi-Layer Picture of Neurodegenerative Diseases: Lessons from the Use of Big Data through Artificial Intelligence. J. Personal. Med..

[51] Lim AC Y., Natarajan P., Fonseka R.D., Maharaj M., Mobbs R.J. (2022). The application of artificial intelligence and custom algorithms with inertial wearable devices for gait analysis and detection of gait-altering pathologies in adults: A scoping review of literature. Digit. Health.

[52] Xu J., Zhang M. (2019). Use of magnetic resonance imaging and artificial intelligence in studies of diagnosis of Parkinson’s disease. ACS Chem. Neurosci..

[53] Zhang Z., Li G., Xu Y., Tang X. (2021). Application of artificial intelligence in the MRI classification task of human brain neurological and psychiatric diseases: A scoping review. Diagnostics.

[54] Yadav D., Garg R.K., Chhabra D., Yadav R., Kumar A., Shukla P. (2020). Smart diagnostics devices through artificial intelligence and mechanobiological approaches. 3 Biotech.

[55] Patil A.D., Biousse V., Newman N.J. (2022). Artificial intelligence in ophthalmology: An insight into neurodegenerative disease. Curr. Opin. Ophthalmol..

[56] Suri J.S., Paul S., Maindarkar M.A., Puvvula A., Saxena S., Saba L., Turk M., Laird J.R., Khanna N.N., Viskovic K. (2022). Cardiovascular/Stroke Risk Stratification in Parkinson’s Disease Patients Using Atherosclerosis Pathway and Artificial Intelligence Paradigm: A Systematic Review. Metabolites.

[57] Vitale A., Villa R., Ugga L., Romeo V., Stanzione A., Cuocolo R. (2021). Artificial intelligence applied to neuroimaging data in Parkinsonian syndromes: Actuality and expectations. Math. Biosci. Eng..

[58] Cascianelli S., Scialpi M., Amici S., Forini N., Minestrini M., Fravolini M., Sinzinger H., Schillaci O., Palumbo B. (2017). Role of artificial intelligence techniques (automatic classifiers) in molecular imaging modalities in neurodegenerative diseases. Curr. Alzheimer Res..

[59] Rana A., Dumka A., Singh R., Panda M.K., Priyadarshi N., Twala B. (2022). Imperative Role of Machine Learning Algorithm for Detection of Parkinson’s Disease: Review, Challenges and Recommendations. Diagnostics.

[60] Raghavendra U., Acharya U.R., Adeli H. (2019). Artificial intelligence techniques for automated diagnosis of neurological disorders. Eur. Neurol..

[61] Singh A.V., Chandrasekar V., Janapareddy P., Mathews D.E., Laux P., Luch A., Yang Y., Garcia-Canibano B., Balakrishnan S., Abinahed J. (2021). Emerging application of nanorobotics and artificial intelligence to cross the BBB: Advances in design, controlled maneuvering, and targeting of the barriers. ACS Chem. Neurosci..

[62] Maitin A.M., Romero Muñoz J.P., García-Tejedor Á.J. (2022). Survey of Machine Learning Techniques in the Analysis of EEG Signals for Parkinson’s Disease: A Systematic Review. Appl. Sci..

[63] Vatansever S., Schlessinger A., Wacker D., Kaniskan H.Ü., Jin J., Zhou M.M., Zhang B. (2021). Artificial intelligence and machine learning-aided drug discovery in central nervous system diseases: State-of-the-arts and future directions. Med. Res. Rev..

[64] Hansen C., Sanchez-Ferro A., Maetzler W. (2018). How mobile health technology and electronic health records will change care of patients with Parkinson’s disease. J. Parkinson’s Dis..

[65] Luis-Martínez R., Monje M.H., Antonini A., Sánchez-Ferro Á., Mestre T.A. (2020). Technology-enabled care: Integrating multidisciplinary care in Parkinson’s disease through digital technology. Front. Neurol..

[66] Fiandaca M.S., Lonser R.R., Elder J.B., Ząbek M., Bankiewicz K.S. (2020). Advancing gene therapies, methods, and technologies for Parkinson’s disease and other neurological disorders. Neurol. Neurochir. Pol..

[67] Kubota K.J., Chen J.A., Little M.A. (2016). Machine learning for large-scale wearable sensor data in Parkinson’s disease: Concepts, promises, pitfalls, and futures. Mov. Disord..

[68] Rana A., Rawat A.S., Bijalwan A., Bahuguna H. Application of multi-layer (perceptron) artificial neural network in the diagnosis system: A systematic review. Proceedings of the 2018 International Conference on Research in Intelligent and Computing in Engineering (RICE).

[69] Baduge S.K., Thilakarathna S., Perera J.S., Arashpour M., Sharafi P., Teodosio B., Mendis P. (2022). Artificial intelligence and smart vision for building and construction 4.0: Machine and deep learning methods and applications. Autom. Constr..

[70] Cunningham P., Cord M., Delany S.J. (2008). Supervised learning. Machine Learning Techniques for Multimedia.

[71] Li N., Shepperd M., Guo Y. (2020). A systematic review of unsupervised learning techniques for software defect prediction. Inf. Softw. Technol..

[72] Czech J. (2021). Distributed methods for reinforcement learning survey. Reinforcement Learning Algorithms: Analysis and Applications.

[73] Esteva A., Robicquet A., Ramsundar B., Kuleshov V., DePristo M., Chou K., Dean J. (2019). A guide to deep learning in healthcare. Nat. Med..

[74] Janiesch C., Zschech P., Heinrich K. (2021). Machine learning and deep learning. Electron. Mark..

[75] Pereira C., Pereira D., Papa J., Rosa G., Yang X. (2016). Convolutional Neural Networks Applied for Parkinson’s Disease Identification. InMachine Learning for Health Informatics.

[76] Shaban M. Deep Convolutional Neural Network for Parkinson’s Disease Based Handwriting Screening. Proceedings of the IEEE International Symposium on Biomedical Imaging.

[77] Frid A., Kantor A., Svechin D., Manevitz L. Diagnosis of Parkinson’s Disease from Continuous Speech Using Deep Convolutional Networks without Manual Selection of Features. Proceedings of the IEEE International Conference on the Science of Electrical Engineering.

[78] Charalambous C. (1992). Conjugate gradient algorithm for efficient training of artificial neural networks. IEE Proc. G (Circuits Devices Syst.).

[79] O’Shea K., Nash R. (2015). An introduction to convolutional neural networks. arXiv.

[80] Saba L., Biswas M., Kuppili V., Godia E.C., Suri H.S., Edla D.R., Omerzu T., Laird J.R., Khanna N.N., Mavrogeni S. (2019). The present and future of deep learning in radiology. Eur. J. Radiol..

[81] Rao K.M.M., Reddy M.S.N., Teja V.R., Krishnan P., Aravindhar D.J., Sambath M. Parkinson’s Disease Detection Using Voice and Spiral Drawing Dataset. Proceedings of the 2020 Third International Conference on Smart Systems and Inventive Technology (ICSSIT).

[82] Jena B., Saxena S., Nayak G.K., Saba L., Sharma N., Suri J.S. (2021). Artificial intelligence-based hybrid deep learning models for image classification: The first narrative review. Comput. Biol. Med..

[83] Raees P.C.M., Thomas V. (2021). Automated detection of Alzheimer’s Disease using Deep Learning in MRI. J. Phys. Conf. Ser..

[84] Oriol J.D.V., Vallejo E.E., Estrada K., Taméz Peña J.G., Disease Neuroimaging Initiative (2019). Benchmarking machine learning models for late-onset alzheimer’s disease prediction from genomic data. BMC Bioinform..

[85] Suri J.S., Agarwal S., Gupta S.K., Puvvula A., Viskovic K., Suri N., Alizad A., El-Baz A., Saba L., Fatemi M. (2021). Systematic Review of Artificial Intelligence in Acute Respiratory Distress Syndrome for COVID-19 Lung Patients: A Biomedical Imaging Perspective. IEEE J. Biomed. Health Inform..

[86] Perlmutter J.S. (2009). Assessment of Parkinson disease manifestations. Curr. Protoc. Neurosci..

[87] Rissardo J.P., Caprara A.F. (2020). Parkinson’s disease rating scales: A literature review. Ann. Mov. Disord..

[88] Samantaray T., Saini J., Gupta C.N. Meta-Analysis of Clinical Symptoms and Data Driven Subtyping Approaches in Parkinson’s Disease. Proceedings of the Brain Conference 2021.

[89] Mohammed F., He X., Lin Y. (2020). An easy-to-use deep-learning model for highly accurate diagnosis of Parkison’s disease using SPECT images. Comput. Med. Imaging Graph..

[90] Sveinbjornsdottir S. (2016). The clinical symptoms of Parkinson’s disease. J. Neurochem..

[91] Bikias T., Iakovakis D., Hadjidimitriou S., Charisis V., Hadjileontiadis L.J. (2021). Deepfog: An IMU-based detection of freezing of gait episodes in Parkison’s disease patients via deep learning. Front. Robot. AI.

[92] Samantaray T., Saini J., Gupta C.N. (2022). Subgrouping and Structural Brain Connectivity of Parkinson’s Disease—Past Studies and Future Directions. Neurosci. Inform..

[93] Morris M., Iansek R., Matyas T., Summers J. (1998). Abnormalities in the stride length-cadence relation in parkinsonian gait. Mov. Disord..

[94] Aita J.F. (1982). Why patients with Parkinson’s disease fall. JAMA.

[95] Koller W.C., Glatt S., Vetere-Overfield B., Hassanein R. (1989). Falls and Parkinson’s disease. Clin. Neuropharmacol..

[96] Morris M.E., Iansek R., Matyas T.A., Summers J.J. (1996). Stride length regulation in Parkinson’s disease. Normalization strategies and underlying mechanisms. Brain.

[97] Hausdorff J.M., Cudkowicz M.E., Firtion R., Wei J.Y., Goldberger A.L. (1998). Gait variability and basal ganglia disorders: Stride-to-stride variations of gait cycle timing in Parkinson’s disease and Huntington’s disease. Mov. Disord..

[98] Vieregge P., Stolze H., Klein C., Heberlein I. (1997). Gait quantitation in Parkinson’s disease—Locomotor disability and correlation to clinical rating scales. Jo. Neural Transm..

[99] Zijlstra W., Rutgers A.W., Van Weerden T.W. (1998). Voluntary and involuntary adaptation of gait in Parkinson’s disease. Gait Posture.

[100] Dashtipour K., Tafreshi A., Lee J., Crawley B. (2018). Speech disorders in Parkinson’s disease: Pathophysiology, medical management and surgical approaches. Neurodegener. Dis. Manag..

[101] https://www.parkinson.org/understanding-parkinsons/symptoms/non-movement-symptoms/small-handwriting.

[102] https://www.healthline.com/health/parkinsons/parkinsons-mri.

[103] https://www.aans.org/en/Patients/Neurosurgical-Conditions-and-Treatments/Parkinsons-Disease.

[104] Iarkov A., Barreto G.E., Grizzell J.A., Echeverria V. (2020). Strategies for the Treatment of Parkinson’s Disease: Beyond Dopamine. Front. Aging Neurosci..

[105] Salamon A., Zádori D., Szpisjak L., Klivényi P., Vécsei L. (2022). What is the impact of catechol-O-methyltransferase (COMT) on Parkinson’s disease treatment? InExpert Opinion on Pharmacotherapy.

[106] Gallazzi M., Mauri M., Bianchi M.L., Riboldazzi G., Cariddi L.P., Carimati F., Rebecchi V., Versino M. (2021). Selegiline reduces daytime sleepiness in patients with Parkinson’s disease. Brain Behav..

[107] Marzoughi S., Banerjee A., Jutzeler C.R., Prado M.A., Rosner J., Cragg J.J., Cashman N. (2021). Tardive neurotoxicity of anticholinergic drugs: A review. J. Neurochem..

[108] Marmol S., Feldman M., Singer C., Margolesky J. (2021). Amantadine Revisited: A Contender for Initial Treatment in Parkinson’s disease?. CNS Drugs.

[109] Avuçlu E., Elen A. (2020). Evaluation of train and test performance of machine learning algorithms and Parkinson diagnosis with statistical measurements. Med. Biol. Eng. Comput..

[110] KarimiRouzbahani H., Daliri M.R. (2011). Diagnosis of Parkinson’s Disease in Human Using Voice Signals. BCN.

[111] Khamparia A., Gupta D., Nguyen N.G., Khanna A., Pandey B., Tiwari P. (2019). Sound Classification Using Convolutional Neural Network and Tensor Deep Stacking Network. IEEE Access.

[112] Bourouhou A., Jilbab A., Nacir C., Hammouch A. Comparison of classification methods to detect the parkinson disease. Proceedings of the 2016 International Conference on Electrical and Information Technologies (ICEIT).

[113] Sharma A., Giri R.N. (2014). Automatic Recognition of Parkinson’s Disease via Artificial Neural Network and Support Vector Machine. Int. J. Innov. Technol. Explor. Eng. (IJITEE).

[114] Purwins H., Li B., Virtanen T., Schluter J., Chang S., Sainath T. (2019). Deep Learning for Audio Signal Processing. IEEE J. Select. Top.Signal Process..

[115] Zhang L., Qu Y., Jin B., Jing L., Gao Z., Liang Z. (2020). An intelligent mobile-enabled system for diagnosing Parkinson disease: Development and validation of a speech impairment detection system. JMIR Med. Inform..

[116] Kadiri S.R., Kethireddy R., Alku P. Parkinson’s disease detection from speech using single frequency filtering cepstral Coefficients. Proceedings of the Interspeech.

[117] Pramanik M., Pradhan R., Nandy P., Bhoi A.K., Barsocchi P. (2021). Machine learning methods with decision forests for Parkinson’s detection. Appl. Sci..

[118] Gunduz H. (2019). Deep Learning-Based Parkinson’s Disease Classification Using Vocal Feature Sets. IEEE Access.

[119] https://www.anjusoftware.com/about/all-news/ai-clinical-trials.

[120] https://www.dataversity.net/improving-clinical-insights-machine-learning/#.

[121] Sakar C.O., Serbes G., Gunduz A., Tunc H.C., Nizam H., Sakar B.E., Tutuncu M., Aydin T., Isenkul M.E., Apaydin H. (2019). A comparative analysis of speech signal processing algorithms for Parkinson’s disease classification and the use of the tunable-factor wavelet transform. Appl. Soft Comput..

[122] Yasar A., Saritas I., Sahman M.A., Cinar A.C. (2019). Classification of Parkinson disease data with artificial neural networks. IOP Conference Series: Materials Science and Engineering.

[123] Ouhmida A., Raihani A., Cherradi B., Terrada O. (2021). A Novel Approach for Parkinson’s Disease Detection Based on Voice Classification and Features Selection Techniques. International Journal of Online & Biomedical Engineering. Int. J. Online Biomed. Eng..

[124] Marar S., Swain D., Hiwarkar V., Motwani N., Awari A. Predicting the occurrence of Parkinson’s Disease using various Classification Models. Proceedings of the 2018 International Conference on Advanced Computation and Telecommunication (ICACAT).

[125] Sheibani R., Nikookar E., Alavi S.E. (2019). An Ensemble Method for Diagnosis of Parkinson’s Disease Based on Voice Measurements. J. Med. Signals Sens..

[126] Tracy J.M., Özkanca Y., Atkins D.C., Ghomi R.H. (2020). Investigating voice as a biomarker: Deep phenotyping methods for early detection of Parkinson’s disease. J. Biomed. Inform..

[127] Cibulka M., Brodnanova M., Grendar M., Grofik M., Kurca E., Pilchova I., Osina O., Tatarkova Z., Dobrota D., Kolisek M. (2019). SNPs rs11240569, rs708727, and rs823156 in SLC41A1 Do Not Discriminate between Slovak Patients with Idiopathic Parkinson’s Disease and Healthy Controls: Statistics and Machine-Learning Evidence. Int. J. Mol. Sci..

[128] Hsu S.-Y., Lin H.-C., Chen T.-B., Du W.-C., Hsu Y.-H., Wu Y.-C., Tu P.-W., Huang Y.-H., Chen H.-Y. (2019). Feasible Classified Models for Parkinson Disease from 99mTc-TRODAT-1 SPECT Imaging. Sensors.

[129] Drotár P., Mekyska J., Rektorová I., Masarová L., Smékal Z., Faundez-Zanuy M. (2016). Evaluation of handwriting kinematics and pressure for differential diagnosis of Parkinson’s disease. Artif. Intell. Med..

[130] Maass F., Michalke B., Willkommen D., Leha A., Schulte C., Tönges L., Mollenhauer B., Trenkwalder C., Rückamp D., Börger M. (2020). Elemental fingerprint: Reassessment of a cerebrospinal fluid biomarker for Parkinson’s disease. Neurobiol. Dis..

[131] Mucha J., Mekyska J., Faundez-Zanuy M., Lopez-De-Ipina K., Zvoncak V., Galaz Z., Kiska T., Smekal Z., Brabenec L., Rektorova I. Advanced Parkinson’s Disease Dysgraphia Analysis Based on Fractional Derivatives of Online Handwriting. Proceedings of the 2018 10th International Congress on Ultra Modern Telecommunications and Control Systems and Workshops (ICUMT).

[132] Wenzel M., Milletari F., Krüger J., Lange C., Schenk M., Apostolova I., Klutmann S., Ehrenburg M., Buchert R. (2019). Automatic classification of dopamine transporter SPECT: Deep convolutional neural networks can be trained to be robust with respect to variable image characteristics. Eur. J. Nucl. Med. Mol. Imaging.

[133] Segovia F., Górriz J.M., Ramírez J., Martínez-Murcia F.J., Castillo-Barnes D. (2019). Assisted diagnosis of Parkinsonism based on the striatal morphology. Int. J. Neural Syst..

[134] Ye Q., Xia Y., Yao Z. (2018). Classification of gait patterns in patients with neurodegenerative disease using adaptive neuro-fuzzy inference system. Comput. Math. Methods Med..

[135] Klomsae A., Auephanwiriyakul S., Theera-Umpon N. (2018). String grammar unsupervised possibilistic fuzzy c-medians for gait pattern classification in patients with neurodegenerative diseases. Comput. Intell. Neurosci..

[136] Felix J.P., Vieira F.H.T., Cardoso A.A., Ferreira M.V.G., Franco R.A.P., Ribeiro M.A., Araujo S.G., Correa H.P., Carneiro M.L. A Parkinson’s Disease Classification Method: An Approach Using Gait Dynamics and Detrended Fluctuation Analysis. Proceedings of the 2019 IEEE Canadian Conference of Electrical and Computer Engineering (CCECE).

[137] Andrei A.-G., Tăuțan A.-M., Ionescu B. Parkinson’s Disease Detection from Gait Patterns. Proceedings of the 2019 E-Health and Bioengineering Conference (EHB).

[138] Priya S.J., Rani A.J., Subathra M.S.P., Mohammed M.A., Damaševičius R., Ubendran N. (2021). Local Pattern Transformation Based Feature Extraction for Recognition of Parkinson’s Disease Based on Gait Signals. Diagnostics.

[139] Yurdakul O.C., Subathra M., George S.T. (2020). Detection of Parkinson’s Disease from gait using Neighborhood Representation Local Binary Patterns. Biomed. Signal Process. Control.

[140] Li B., Yao Z., Wang J., Wang S., Yang X., Sun Y. (2020). Improved Deep Learning Technique to Detect Freezing of Gait in Parkinson’s Disease Based on Wearable Sensors. Electronics.

[141] Gong B., Shi J., Ying S., Dai Y., Zhang Q., Dong Y., Zhang Y. (2018). Neuroimaging-based diagnosis of Parkinson’s disease with deep neural mapping large margin distribution machine. Neurocomputing.

[142] Chakraborty S., Aich S., Kim H.-C. (2020). 3D textural, morphological and statistical analysis of voxel of interests in 3T MRI scans for the detection of Parkinson’s disease using artificial neural networks. Healthcare.

[143] Bhan A., Kapoor S., Gulati M., Goyal A. Early diagnosis of Parkinson’s disease in brain MRI using deep learning algorithm. Proceedings of the 2021 Third International Conference on Intelligent Communication Technologies and Virtual Mobile Networks.

[144] Kumar R., Gupta A., Arora H.S., Raman B. (2021). IBRDM: An intelligent framework for brain tumor classification using radiomics-and DWT-based fusion of MRI sequences. ACM Trans. Internet Technol. (TOIT).

[145] Pang Y., Christenson J., Jiang F., Lei T., Rhoades R., Kern D., Thompson J.A., Liu C. (2020). Automatic detection and quantification of hand movements toward development of an objective assessment of tremor and bradykinesia in Parkison’s disease. J. Neurosci. Methods.

[146] Radziunas A., Deltuva V.P., Tamasauskas A., Gleizniene R., Pranckeviciene A., Petrikonis K., Bunevicius A. (2018). Brain MRI morphometric analysis in Parkinson’s disease patients with sleep disturbances. BMC Neurol..

[147] Zhang D., Wang J., Liu X., Chen J., Liu B. (2015). Aberrant brain network efficiency in Parkinson’s disease patients with tremor: A multi-modality study. Front. Aging Neurosci..

[148] Kiryu S., Yasaka K., Akai H., Nakata Y., Sugomori Y., Hara S., Seo M., Abe O., Ohtomo K. (2019). Deep learning to differentiate parkinsonian disorders separately using single midsagittal MR imaging: A proof of concept study. Eur. Radiol..

[149] Magesh P.R., Myloth R.D., Tom R.J. (2020). An explainable machine learning model for early detection of Parkinson’s disease using LIME on DaTSCAN imagery. Comput. Biol. Med..

[150] Mabrouk R., Chikhaoui B., Bentabet L. (2018). Machine learning based classification using clinical and DaTSCAN SPECT imaging features: A study on Parkinson’s disease and SWEDD. IEEE Trans. Radiat. Plasma Med. Sci..

[151] Quan J., Xu L., Xu R., Tong T., Su J. (2019). DaTscan SPECT Image Classification for Parkinson’s Disease. arXiv.

[152] Moon S., Song H.J., Sharma V.D., Lyons K.E., Pahwa R., Akinwuntan A.E., Devos H. (2020). Classification of Parkinson’s disease and essential tremor based on balance and gait characteristics from wearable motion sensors via machine learning techniques: A data-driven approach. J. Neuroeng. Rehabil..

[153] Adams M.P., Rahmim A., Tang J. (2021). Improved motor outcome prediction in Parkinson’s disease applying deep learning to DaTscan SPECT images. Comput. Biol. Med..

[154] Khachnaoui H., Khlifa N., Mabrouk R. (2022). Machine Learning for Early Parkinson’s Disease Identification within SWEDD Group Using Clinical and DaTSCAN SPECT Imaging Features. J. Imaging.

[155] Oliveira F.P., Faria D.B., Costa D.C., Castelo-Branco M., Tavares J.M.R. (2018). Extraction, selection and comparison of features for an effective automated computer-aided diagnosis of Parkinson’s disease based on [123I] FP-CIT SPECT images. Eur. J. Nucl. Med. Mol. Imaging.

[156] Saponaro S., Giuliano A., Bellotti R., Lombardi A., Tangaro S., Oliva P., Retico A. (2022). Multi-site harmonization of MRI data uncovers machine-learning discrimination capability in barely separable populations: An example from the ABIDE dataset. NeuroImage Clin..

[157] Tufail A.B., Ma Y.K., Zhang Q.N., Khan A., Zhao L., Yang Q., Adeel M., Khan R., Ullah I. (2021). 3D convolutional neural networks-based multiclass classification of Alzheimer’s and Parkinson’s diseases using PET and SPECT neuroimaging modalities. Brain Inform..

[158] Antikainen E., Cella P., Tolonen A., van Gils M. SPECT Image Features for Early Detection of Parkinson’s Disease using Machine Learning Methods. Proceedings of the 2021 43rd Annual International Conference of the IEEE Engineering in Medicine & Biology Society (EMBC).

[159] Salvatore C., Cerasa A., Castiglioni I., Gallivanone F., Augimeri A., Lopez M., Quattrone A. (2014). Machine learning on brain MRI data for differential diagnosis of Parkinson’s disease and Progressive Supranuclear Palsy. J. Neurosci. Methods.

[160] Martínez-Ibañez M., Ortiz A., Munilla J., Salas-Gonzalez D., Górriz J.M., Ramírez J. (2019). Isosurface modelling of DatSCAN images for parkinson disease diagnosis. International Work-Conference on the Interplay between Natural and Artificial Computation.

[161] Kurmi A., Biswas S., Sen S., Sinitca A., Kaplun D., Sarkar R. (2022). An Ensemble of CNN Models for Parkinson’s Disease Detection Using DaTscan Images. Diagnostics.

[162] Hossen A. (2013). A neural network approach for feature extraction and discrimination between parkinsonian tremor and essential tremor. Technol. Health Care.

[163] Challa KN R., Pagolu V.S., Panda G., Majhi B. An improved approach for prediction of Parkison’s disease using machine learning techniques. Proceedings of the 2016 international conference on signal processing, communication, power and embedded system (SCOPES).

[164] Choi H., Ha S., Im H.J., Paek S.H., Lee D.S. (2017). Refining diagnosis of Parkinson’s disease with deep learning-based interpretation of dopamine transporter imaging. NeuroImage Clin..

[165] Kim D.H., Wit H., Thurston M. (2018). Artificial intelligence in the diagnosis of Parkison’s disease from ioflupane-123 single-photon emission computed tomography dopamine transporter scans using transfer learning. Nucl. Med. Commun..

[166] Esmaeilzadeh S., Yang Y., Adeli E. (2018). End-to-end parkinson disease diagnosis using brain MR-images by 3D-CNN. arXiv.

[167] Kim H.B., Lee W.W., Kim A., Lee H.J., Park H.Y., Jeon H.S., Kim S.K., Jeon B., Park K.S. (2018). Wrist sensor-based tremor severity quantification in Parkison’s disease using convolutional neural network. Comput. Biol. Med..

[168] Martinez-Murcia F.J., Ortiz A., Górriz J.M., Ramírez J., Segovia F., Salas-Gonzalez D., Castillo-Barnes D., Illán I.A. (2017). A 3D convolutional neural network approach for the diagnosis of Parkison’s disease. International Work-Conference on the Interplay between Natural and Artificial Computation.

[169] Qin Z., Jiang Z., Chen J., Hu C., Ma Y. (2019). SEMG-based tremor severity evaluation for Parkison’s disease using a light-weight CNN. IEEE Signal Process. Lett..

[170] Kollia I., Stafylopatis A.-G., Kollias S. Predicting Parkison’s disease using latent information extracted from deep neural networks. Proceedings of the 2019 International Joint Conference on Neural Networks.

[171] Szumilas M., Lewenstein K., Ślubowska E., Szlufik S., Koziorowski D. (2020). A multimodal approach to the quantification of kinetic tremor in Parkison’s disease. Sensors.

[172] Oktay A.B., Kocer A. (2020). Differential diagnosis of Parkinson and essential tremor with convolutional LSTM networks. Biomed. Signal Process. Control.

[173] Shahtalebi S., Atashzar S.F., Samotus O., Patel R.V., Jog M.S., Mohammadi A. (2020). Phtnet: Characterization and deep mining of involuntary pathological hand tremor using recurrent neural network models. Sci. Rep..

[174] Veeraragavan S., Gopalai A.A., Gouwanda D., Ahmad S.A. (2020). Parkison’s disease diagnosis and severity assessment using ground reaction forces and neural networks. Front. Physiol..

[175] Chien C.-Y., Hsu S.-W., Lee T.-L., Sung P.-S., Lin C.-C. (2021). Using artificial neural network to discriminate Parkison’s disease from other parkinsonisms by focusing on putamen of dopamine transporter SPECT images. Biomedicines.

[176] Yasaka K., Kamagata K., Ogawa T., Hatano T., Takeshige-Amano H., Ogaki K., Andica C., Akai H., Kunimatsu A., Uchida W. (2021). Parkison’s disease: Deep learning with a parameter-weighted structural connectome matrix for diagnosis and neural circuit disorder investigation. Neuroradiology.

[177] Yang Y., Wei L., Hu Y., Wu Y., Hu L., Nie S. (2021). Classification of Parkison’s disease based on multi-modal features and stacking ensemble learning. J. Neurosci. Methods.

[178] Vyas T., Yadav R., Solanki C., Darji R., Desai S., Tanwar S. (2022). Deep learning-based scheme to diagnose Parkison’s disease. Expert Syst..

[179] Yadav S. (2021). Bayesian Deep Learning Based Convolutional Neural Network for Classification of Parkison’s Disease Using Functional Magnetic Resonance Images. https://papers.ssrn.com/sol3/papers.cfm?abstract_id=3833760.

[180] Mei J., Desrosiers C., Frasnelli J. (2021). Machine Learning for the Diagnosis of Parkinson’s disease: A Review of Literature. Front. Aging Neurosci..

[181] Khedr E.M., El Fetoh N.A., Khalifa H.E., Ahmed M.A., El Beh K.M. (2013). Prevalence of non-motor features in a cohort of Parkinson’s disease patients. Clin. Neurol. Neurosurg..

[182] Zappia M., Annesi G., Nicoletti G., Arabia G., Annesi F., Messina D., Pugliese P., Spadafora P., Tarantino P., Carrideo S. (2005). Sex differences in clinical and genetic determinants of levodopa peak-dose dyskinesias in Parkinson disease: An exploratory study. Arch. Neurol..

[183] Ma X., Niu Y., Gu L., Wang Y., Zhao Y., Bailey J., Lu F. (2021). Understanding adversarial attacks on deep learning based medical image analysis systems. Pattern Recogn..

[184] Alluri R.K., Vaishnav A.S., Sivaganesan A., Ricci L., Sheha E., Qureshi S.A. (2021). Multimodality Intraoperative Neuromonitoring in Lateral Lumbar Interbody Fusion: A Review of Alerts in 628 Patients. Glob. Spine J..

[185] Hassan M., Chaton L., Benquet P., Delval A., Leroy C., Plomhause L., Dujardin K. (2017). Functional connectivity disruptions correlate with cognitive phenotypes in Parkinson’s disease. NeuroImage Clin..

[186] Sadeghi D., Shoeibi A., Ghassemi N., Moridian P., Khadem A., Alizadehsani R., Acharya U.R. (2022). An overview of artificial intelligence techniques for diagnosis of Schizophrenia based on magnetic resonance imaging modalities: Methods, challenges, and future works. Comput. Biol. Med..

[187] Utianski R.L., Caviness J.N., van Straaten E.C., Beach T.G., Dugger B.N., Shill H.A., Hentz J.G. (2016). Graph theory network function in Parkinson’s disease assessed with electroencephalography. Clin. Neurophysiol..

[188] Zhang Y.D., Dong Z., Wang S.H., Yu X., Yao X., Zhou Q., Gorriz J.M. (2020). Advances in multimodal data fusion in neuroimaging: Overview, challenges, and novel orientation. Inf. Fusion.

[189] Vij R., Arora S. (2022). Computer Vision with Deep Learning Techniques for Neurodegenerative Diseases Analysis Using Neuroimaging: A Survey. International Conference on Innovative Computing and Communications.

[190] Xu J., Jiao F., Huang Y., Luo X., Xu Q., Li L., Zhuang X. (2019). A fully automatic framework for parkinson’s disease diagnosis by multi-modality images. Front. Neurosci..

[191] Tăuţan A.M., Ionescu B., Santarnecchi E. (2021). Artificial intelligence in neurodegenerative diseases: A review of available tools with a focus on machine learning techniques. Artif. Intell. Med..

[192] Yao A.D., Cheng D.L., Pan I., Kitamura F. (2020). Deep learning in neuroradiology: A systematic review of current algorithms and approaches for the new wave of imaging technology. Radiol. Artif. Intell..

[193] Hall E.L., Robson S.E., Morris P.G., Brookes M.J. (2014). The relationship between MEG and fMRI. Neuroimage.

[194] Moridian P., Ghassemi N., Jafari M., Salloum-Asfar S., Sadeghi D., Khodatars M., Acharya U.R. (2022). Automatic Autism Spectrum Disorder Detection Using Artificial Intelligence Methods with MRI Neuroimaging: A Review. arXiv.

[195] Zou L., Zheng J., Miao C., Mckeown M.J., Wang Z.J. (2017). 3D CNN based automatic diagnosis of attention deficit hyperactivity disorder using functional and structural MRI. IEEE Access.

[196] Amini M., Pedram M.M., Moradi A., Jamshidi M., Ouchani M. (2021). Single and combined neuroimaging techniques for Alzheimer’s disease detection. Comput. Intell. Neurosci..

[197] Shoeibi A., Ghassemi N., Khodatars M., Moridian P., Khosravi A., Zare A., Acharya U.R. (2022). Automatic Diagnosis of Schizophrenia and Attention Deficit Hyperactivity Disorder in rs-fMRI Modality using Convolutional Autoencoder Model and Interval Type-2 Fuzzy Regression. arXiv.

[198] Kelly C.J., Karthikesalingam A., Suleyman M., Corrado G., King D. (2019). Key challenges for delivering clinical impact with artificial intelligence. BMC Med..

[199] Sivaranjini S., Sujatha C.M. (2020). Deep learning based diagnosis of Parkinson’s disease using convolutional neural network. Multimed. Tools Appl..

[200] Yagis E., De Herrera A.G.S., Citi L. Generalization performance of deep learning models in neurodegenerative disease classification. Proceedings of the 2019 IEEE International Conference on Bioinformatics and Biomedicine (BIBM).

[201] Lee S., Hussein R., McKeown M.J. A deep convolutional-recurrent neural network architecture for Parkinson’s disease EEG classification. Proceedings of the 2019 IEEE Global Conference on Signal and Information Processing (GlobalSIP).

[202] Noor MB T., Zenia N.Z., Kaiser M.S., Mahmud M., Mamun S.A. (2019). Detecting neurodegenerative disease from MRI: A brief review on a deep learning perspective. International Conference on Brain Informatics.

[203] Paré G., Trudel M.C., Jaana M., Kitsiou S. (2015). Synthesizing information systems knowledge: A typology of literature reviews. Inf. Manag..

[204] Li R., Zhang W., Suk H.I., Wang L., Li J., Shen D., Ji S. (2014). Deep learning based imaging data completion for improved brain disease diagnosis. International Conference on Medical Image Computing and Computer-Assisted Intervention.

[205] Nie D., Trullo R., Lian J., Petitjean C., Ruan S., Wang Q., Shen D. (2017). Medical image synthesis with context-aware generative adversarial networks. International Conference on Medical Image Computing and Computer-Assisted Intervention.

[206] Nie D., Trullo R., Lian J., Wang L., Petitjean C., Ruan S., Shen D. (2018). Medical image synthesis with deep convolutional adversarial networks. IEEE Trans. Biomed. Eng..

[207] Cai L., Wang Z., Gao H., Shen D., Ji S. Deep adversarial learning for multi-modality missing data completion. Proceedings of the 24th ACM SIGKDD International Conference on Knowledge Discovery & Data Mining.

[208] Pan Y., Liu M., Lian C., Zhou T., Xia Y., Shen D. (2018). Synthesizing missing PET from MRI with cycle-consistent generative adversarial networks for Alzheimer’s disease diagnosis. International Conference on Medical Image Computing and Computer-Assisted Intervention.

[209] Zhi Y., Wang M., Yuan Y.S., Shen Y.T., Ma K.W., Gan C.T., Si Q.Q., Wang L.N., Cao S.W., Zhang K.Z. (2019). The increased gray matter volumes of precentralgyri in Parkinson’s disease patients with diphasic dyskinesia. Aging (Albany NY).

[210] Li P., Xu P., Liu J. (2021). Biomarkers and Pathogenesis of Alpha-Synuclein in Parkinson’s Disease. Aging Neurosci..

[211] Yuan Y.S., Ji M., Gan C.T., Sun H.M., Wang L.N., Zhang K.Z. (2022). Impaired Interhemispheric Synchrony in Parkinson’s Disease with Fatigue. J. Personal. Med..

[212] Daveau R.S., Law I., Henriksen O.M., Hasselbalch S.G., Andersen U.B., Anderberg L., Højgaard L., Andersen F.L., Ladefoged C.N. (2022). Deep learning based low-activity PET reconstruction of [11C] PiB and [18F] FE-PE2I in neurodegenerative disorders. Neuroimage.

[213] Noor MB T., Zenia N.Z., Kaiser M.S., Mamun S.A., Mahmud M. (2020). Application of deep learning in detecting neurological disorders from magnetic resonance images: A survey on the detection of Alzheimer’s disease, Parkinson’s disease and schizophrenia. Brain inform..

[214] Kang S.K., Seo S., Shin S.A., Byun M.S., Lee D.Y., Kim Y.K., Lee J.S. (2018). Adaptive template generation for amyloid PET using a deep learning approach. Hum. Brain Mapp..

[215] Liu M., Cheng D., Wang K., Wang Y. (2018). Multi-modality cascaded convolutional neural networks for Alzheimer’s disease diagnosis. Neuroinformatics.

[216] Oh K., Chung Y.C., Kim K.W., Kim W.S., Oh I.S. (2019). Classification and visualization of Alzheimer’s disease using volumetric convolutional neural network and transfer learning. Sci. Rep..

[217] Wang S.H., Phillips P., Sui Y., Liu B., Yang M., Cheng H. (2018). Classification of Alzheimer’s disease based on eight-layer convolutional neural network with leaky rectified linear unit and max pooling. J. Med. Syst..

[218] Shang R., Wang J., Jiao L., Yang X., Li Y. (2022). Spatial feature-based convolutional neural network for PolSAR image classification. Appl. Soft Comput..

[219] Ye A., Zhou X., Miao F. (2022). Innovative Hyperspectral Image Classification Approach Using Optimized CNN and ELM. Electronics.

[220] Mohapatra M., Parida A.K., Mallick P.K., Zymbler M., Kumar S. (2022). Botanical Leaf Disease Detection and Classification Using Convolutional Neural Network: A Hybrid Metaheuristic Enabled Approach. Computers.

[221] Hei Y., Liu C., Li W., Ma L., Lan M. (2022). CNN Based Hybrid Precoding for MmWave MIMO Systems with Adaptive Switching Module and Phase Modulation Array. IEEE Trans. Wirel. Commun..

[222] Aslan M.F., Unlersen M.F., Sabanci K., Durdu A. (2021). CNN-based transfer learning–BiLSTM network: A novel approach for COVID-19 infection detection. Appl. Soft Comput..

